# Sex-specific epigenetics drive low GPER expression in gastrointestinal smooth muscles in type 2 diabetic mice

**DOI:** 10.1038/s41598-024-54213-7

**Published:** 2024-03-07

**Authors:** Aliyu Muhammad, Juanita C. Hixon, Abdurrahman Pharmacy Yusuf, Jatna I. Rivas Zarete, India Johnson, Jamial Miller, Benjamin Adu-Addai, Clayton Yates, Sunila Mahavadi

**Affiliations:** 1 Department of Biology, Center for Cancer Research, Tuskegee University, Tuskegee, AL 36088 USA; 2 Department of Biochemistry, Faculty of Life Sciences, Ahmadu Bello University, P.M.B. 1044, Zaria, Kaduna State Nigeria; 3 Department of Biochemistry, Federal University of Technology, P.M.B 65, Minna, Niger State Nigeria; 4 Department of Biomedical Sciences, College of Veterinary Medicine, Tuskegee University, Tuskegee, AL 36088 USA; 5 Department of Pathology, Johns Hopkins University School of Medicine, Baltimore, MD USA

**Keywords:** GPER, Epigenomics, Type 2 diabetes, Smooth muscle, Sex, Molecular biology, Diseases, Gastroenterology

## Abstract

Type 2 diabetes mellitus (T2D) causes gastroparesis, delayed intestinal transit, and constipation, for unknown reasons. Complications are predominant in women than men (particularly pregnant and postmenopausal women), suggesting a female hormone-mediated mechanism. Low G-protein coupled estrogen receptor (GPER) expression from epigenetic modifications may explain it. We explored sexually differentiated GPER expression and gastrointestinal symptoms related to GPER alterations in wild-type (WT) and T2D mice (*db/db*). We also created smooth muscle-specific *GPER* knockout (*GPER* KO) mice to phenotypically explore the effect of GPER deficiency on gastrointestinal motility. GPER mRNA and protein expression, DNA methylation and histone modifications were measured from stomach and colon samples of *db/db* and WT mice*.* Changes in gut motility were also evaluated as daily fecal pellet production patterns. We found that WT female tissues have the highest GPER mRNA and protein expressions. The expression is lowest in all *db/db*. GPER downregulation is associated with promoter hypermethylation and reduced enrichment of H3K4me3 and H3K27ac marks around the GPER promoter. We also observed sex-specific disparities in fecal pellet production patterns of the GPER KO mice compared to WT. We thus, conclude that T2D impairs gut GPER expression, and epigenetic sex-specific mechanisms matter in the downregulation.

## Introduction

Diabetes mellitus (DM) and its complications are among the leading causes of global morbidity and mortality^1^. As of December 2021, the estimated burden of DM hits 537 million cases and over 6 million deaths globally. Type 2 diabetes mellitus (T2D) represents about 90% of the diabetes cases in the USA, predominantly affecting people 45 years old and over^2^. Diabetes-induced gastrointestinal tract (GIT) disorders (particularly diabetic gastroenteropathy) are among the poorly diagnosed long-term sequelae of peripheral and autonomic neuropathy^3–5^. GIT-related DM complications usually affect all cells of the GIT, including the enteric neurons, the tunica muscularis/layers of smooth muscle cells (SMCs), the vascular endothelium within the myenteric plexus ganglia, and the two types of interstitial cells: interstitial cells of Cajal (ICC) and interstitial platelet-derived growth factor receptor α-positive cells (IPC)^5–8^. The pathological changes in these cells caused by DM alter gastrointestinal motility (GIM). The dysregulated GIM consequently leads to upper and lower GIT disorders such as gastroesophageal reflux disease (GERD), delayed gastric emptying (gastroparesis), delayed intestinal transit, and constipation^3,4,9,10^. Smooth muscle contractions in the gastric, colonic, and other regions of the GIT, also known as GIM, are essential for the proper and timely mixing, digestion, movement, absorption, and egestion of food nutrients, which ultimately affects glycemic control in DM^10^. Despite all this, gastroparesis has been shown to be more severe in female than male diabetic rats, attributed to differences in nitric oxide production (greater in females). Nitric oxide production, in turn, is increased by female reproductive hormones in at least some parts of the GI tract, which may help explain the difference in the severity of gastroparesis in diabetes^11,12^.

The importance of female hormones in gastrointestinal motility has been placed on the stage by findings that, when such hormones disappear due to menopause, differences in gastrointestinal motility between men and women practically disappear^13^. Indeed, in premenopause, gastric emptying is slower in females than in males, which is attributed to longer proximal gastric relaxation times and decreased distal stomach contractility^14^.

The question posed by this reality is which hormones, specifically, are determining the differences in GI motility between men and women. This is important because women have a higher risk of GI complications, including gastroparesis and constipation. Estrogens in particular are known to delay gastric emptying^15^, but activate 3 different receptors which may, isolatedly or in conjunction, explain the phenomenon.

The discovery of the novel GPER about 26 years ago has enabled scientists to mechanistically decipher the roles of estrogens and estrogen mimetics in the pathogenesis and management of chronic diseases such as obesity, T2D, and cancer^2,16–18^. Accordingly, gender disparities in GPER expression have also helped elucidate the mechanism behind the additional protection against metabolic diseases that is peculiar to estrogen-rich models like premenopausal females compared to estrogen-deficient models such as males as well as ovariectomized and postmenopausal females^16,19–21^. GPER activation leads to relaxation of GI smooth muscles^22^, and affects fecal pellet deposition in mice, prompting an investigation of its impact in other parts of the GI tract^12,23^. Moreover, differences in GPER expression between the sexes motivated a differentiated investigation of the impact of GPER in the male and female GI tracts.

However, to the best of our knowledge, whether GPER expression and signaling could be involved in the protection against or the pathogenesis of diabetes-induced smooth muscle dysfunction in the GIT via epigenetic modifications is yet to be reported in the existing literature.

Epigenetic modifications such as the 5′-cytosine methylation of DNA (especially the methyl marks deposited on the CpG islands of promoter sequences) and histone tail posttranslational modifications are critical in regulating gene expression^24–28^. These modifications usually work in a coordinated manner to facilitate chromatin remodeling, which causes the activation or suppression of the nearby genes^29^. Gene silencing is generally associated with promoter hypermethylation due to the upregulation of DNA methyltransferases (DNMTs) and reduced enrichment of active histone marks such as histone 3 lysine 4 trimethylations (H3K4me3) and histone 3 lysine 27 acetylation (H3K27ac), which is the hallmark of an inactive chromatin^29,30^.

To our knowledge, previous research has yet to address epigenetic changes and GPER expression in T2D. This is despite the knowledge that *GPER* Knockout (*GPER* KO) mice present with insulin resistance, which is a characteristic of T2D^31^. *GPER* KO mice present with glucose intolerance and a tendency toward T2D^31^. Hence the significance of measuring GPER expression in the T2D mouse model (*db/db* mice), and the epigenetic markers that may further unravel the pathological changes. Moreover, the T2D incidence and complication burden differs between men and women before the onset of menopause, which suggests differences that may be hormonally mediated and that need to be accounted for^31–33^.

Due to the gastrointestinal manifestation of T2D, we decided to explore the GPER expression of a T2D mouse model (*db/db* mice) in the gastric and colonic smooth muscles of *db/db* mice compared to wild-type (WT) mice. We also explored the gender disparities therein. Consequently, we were able to demonstrate that GPER downregulation is associated with promoter hyper-methylation and reduced enrichment of H3K4me3 and H3K27ac marks around the GPER promoter, implying that T2D impairs GPER expression in the gut, and epigenetic mechanisms driven by sex-specific disparity play a significant role in the downregulation.

## Results

### *GPER* mRNA expression is downregulated in the stomach and colon of *db/db* mice

**Figure 1:**
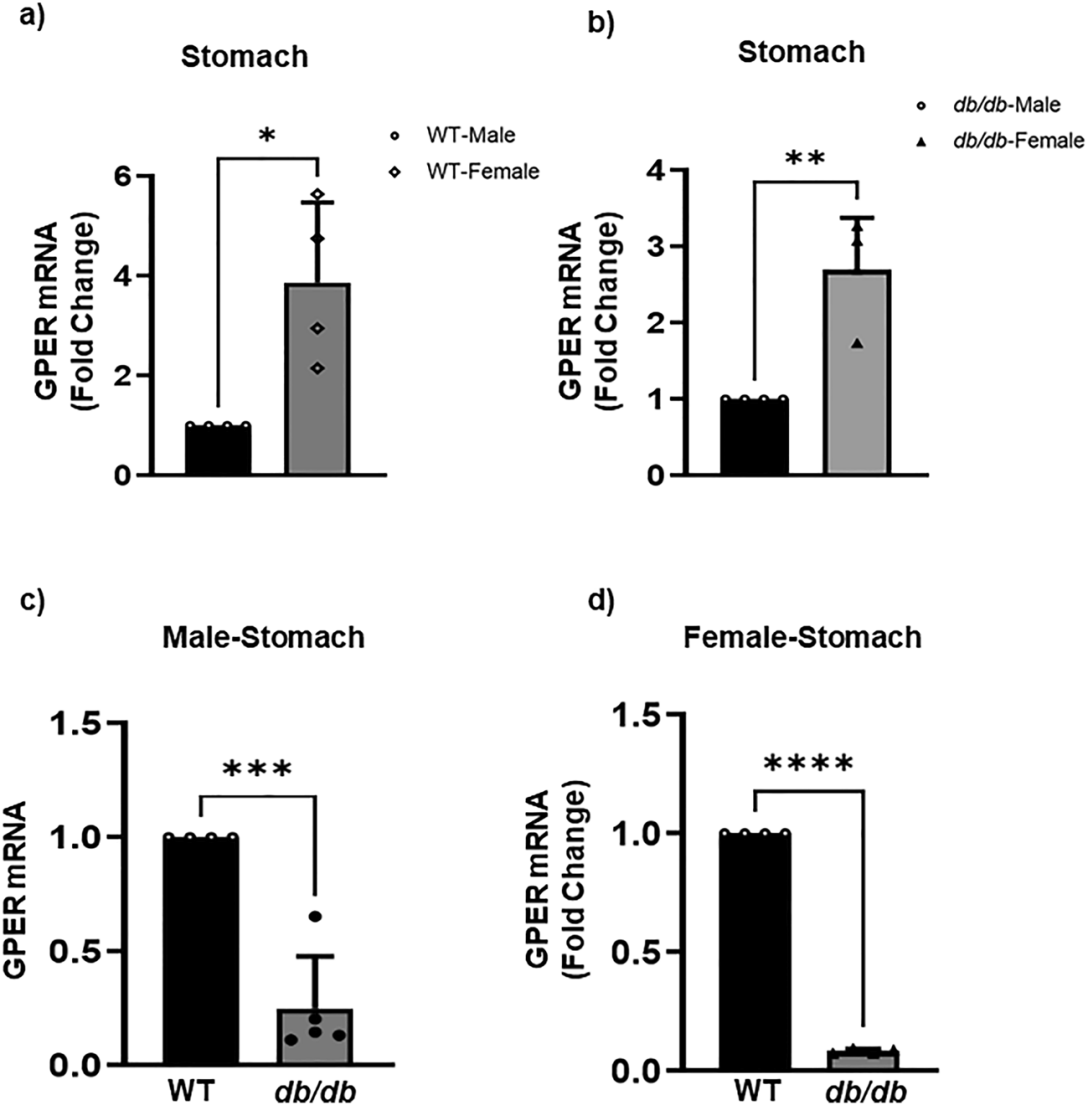
GPER mRNA expression in the gastric smooth muscles. (**a**) qRT-PCR analysis showing GPER mRNA expression (in fold change) of the WT male mice (black bar) versus female mice (gray bar). The bar size represents the mean of n = 5 biologically independent WT animals per group of males and females. Results were deemed significant when p < 0.05. The t-test generated p-value is significant, at *p = 0.016. (**b**) GPER mRNA expression (in fold change) of the *db/db* male (black bar) versus female (gray bar) mice. The bar size represents the mean of n = 5 biologically independent *db/db* animals per group of males and females. The t-test generated p-value is significant, at **p = 0.002. (**c**) GPER mRNA expression (in fold change) of the WT male (black bar) versus *db/db* male (gray bar) mice. The bar size represents the mean of n = 5 biologically independent animals per group of the WT versus *db/db* males. The t-test generated p-value is significant, at ***p = 0.0003. (**d**) GPER mRNA expression (in fold change) of the WT female (black bar) versus *db/db* female (gray bar) mice. The bar size represents the mean of n = 5 biologically independent animals per group of the WT versus *db/db* females. The t-test generated p-value is significant at ****p < 0.0001.

**Figure 2:**
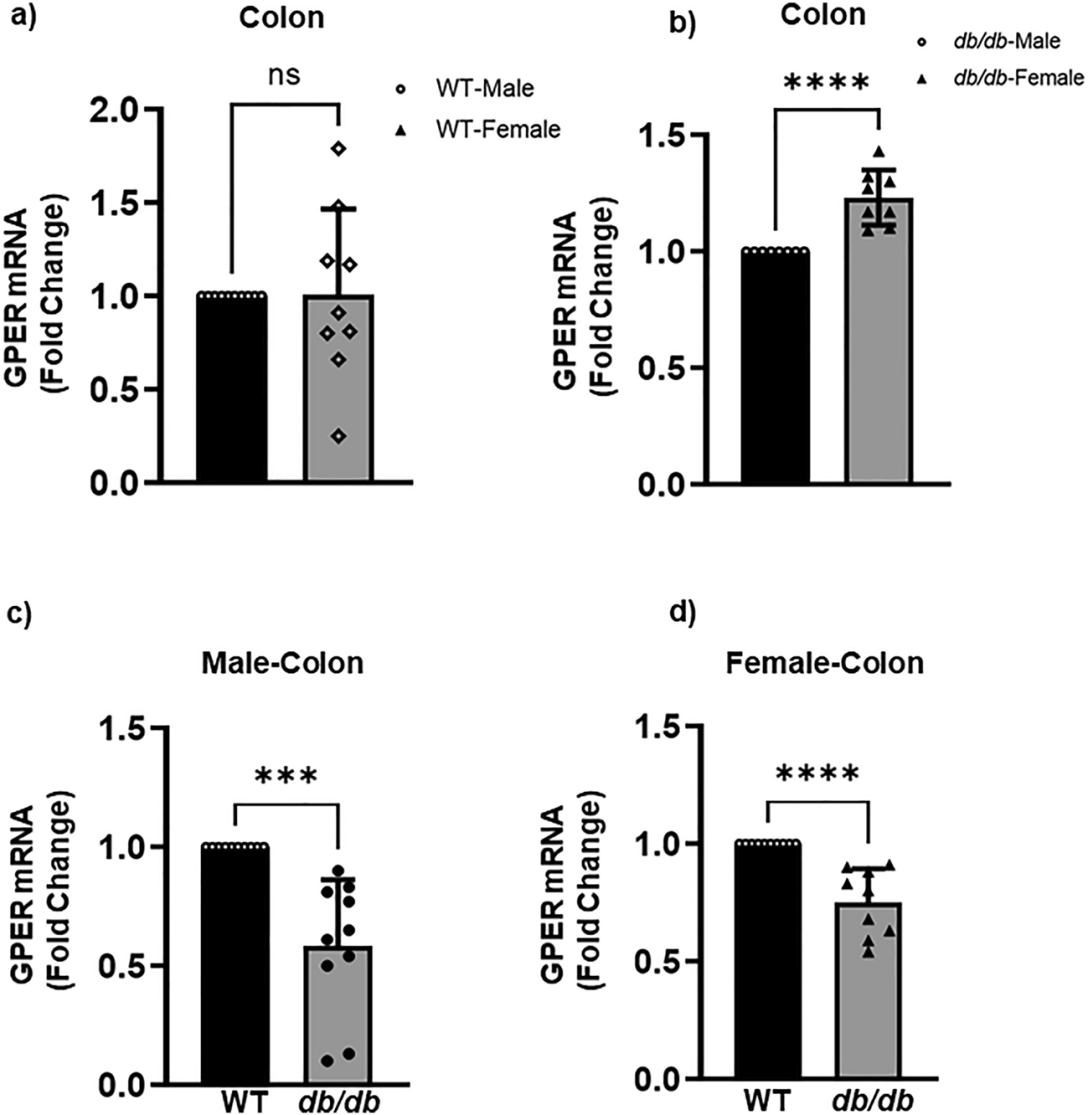
GPER mRNA expression in the colonic smooth muscles. (**a**) qRT-PCR analysis showing GPER mRNA expression (in fold change) of the WT male mice (black bar) versus female mice (gray bar). Results were deemed significant when p < 0.05. The bar size represents the mean of n = 5 biologically independent WT animals per group of males and of females. The black dots on the black bar represent the individual values per mouse in the WT group, and the black triangles on the gray bar represent individual values per mouse in the *db/db* group distributed around the error bar. The t-test generated p-value is significant at *p = 0.02. (**b**) GPER mRNA expression (in fold change) of the WT male (black bar) versus *db/db* male (gray bar). The bar size represents the mean of n = 5 biologically independent animals per group of the WT versus *db/db* males. The t-test generated p-value is significant at ****p < 0.0001. (**c**) GPER mRNA expression (in fold change) of the *db/db* male (black bar) versus *db/db* female (gray bar). The bar size represents the mean of n = 5 biologically independent animals per group of the *db/db* males and females. The t-test generated p-value is significant at ***p = 0.0006.

To check whether there are variations in *GPER* expression in the *db/db* and WT mice, we first measured *GPER* expression at the transcriptomic level. We extracted total mRNA from gastric and colonic smooth muscle tissue strips and subjected it to quantitative real-time PCR (qRT-PCR). In the stomach, we found that females generally exhibited higher *GPER* mRNA expression than males across the WT (286.9 ± 71.8% increase) (Fig. 1a) and *db/db* groups (169.2 ± 37.2% increase) (Fig. 1b). However, in both sexes, the *db/db* mice exhibited significantly lower *GPER* mRNA levels compared to the WT controls by 75 ± 10%, and 92 ± 0.4% (Fig. 1c and d, correspondingly). In the colon, there is no significant difference in *GPER* mRNA expression between WT males and females (Fig. 2a); however there is higher expression in *db/db* females compared to *db/db* males (19 ± 7.1%, difference) (Fig. 2b). In both the colons of male and female *db/db* mice, there was a lower *GPER* mRNA expression when compared to WT male and female mice (36 ± 9%; 19 ± 6.6% decrease), correspondingly (Fig. 2c and d). This result, therefore, implies that T2D-induced alterations in *GPER* mRNA expression differences between the gastric and colonic smooth muscle in a sex-specific manner.

### GPER protein expression is downregulated in the stomach and colon of *db/db* mice

**Figure 3:**
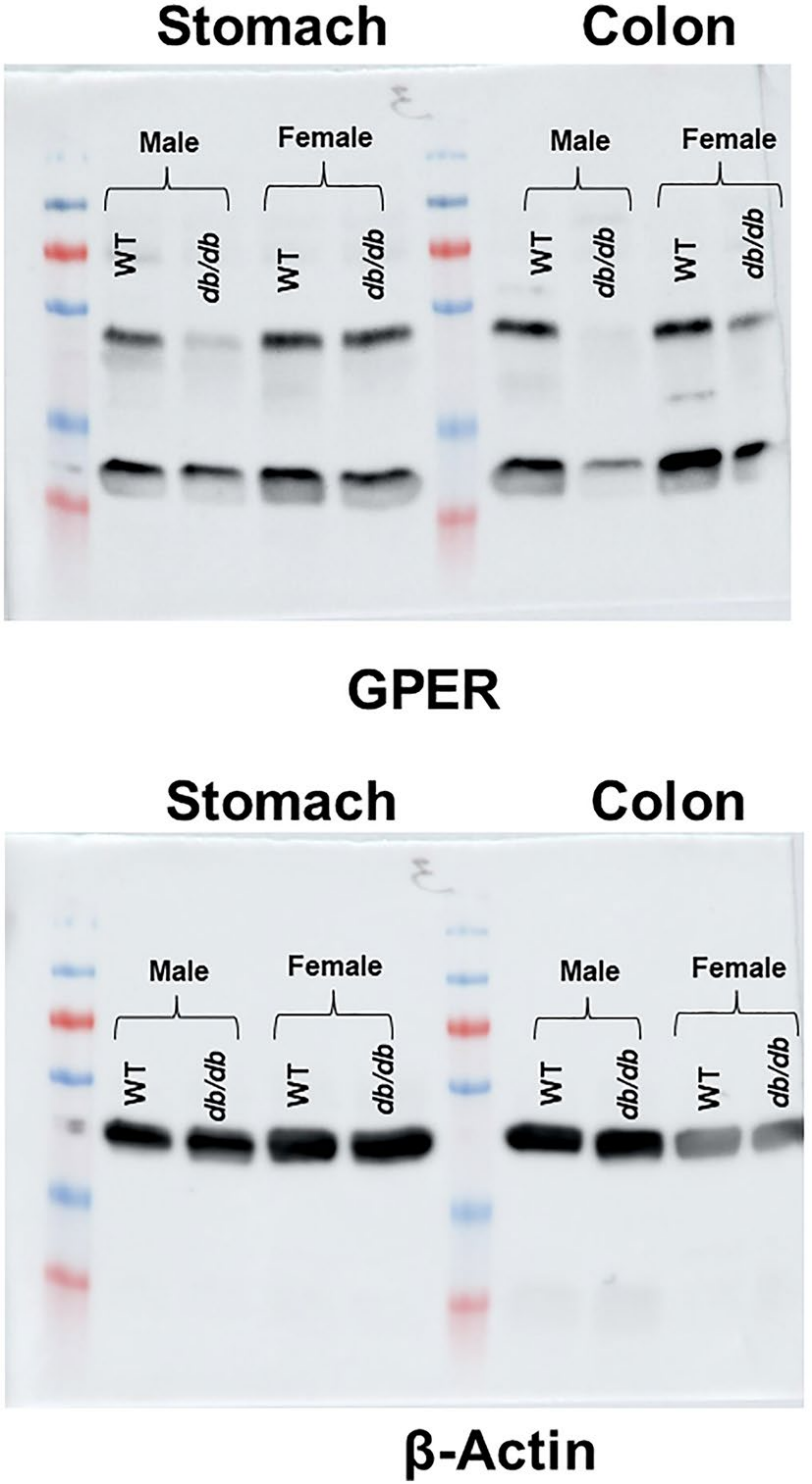
GPER protein expression in the gastric and colonic smooth muscles. Western blot analysis showing the protein content of GPER (left) compared to β-actin (right) in the gastric and colonic smooth muscles of the WT versus *db/db* male and female mice. In the left figure, the first four globular shapes represent the GPER protein bands in the gastric smooth muscle strips of the WT male, *db/db* male, WT female, and *db/db* female respectively. The last four bands represent the same thing for the colonic smooth muscles.

To validate our mRNA data, we conducted a Western blot analysis to measure GPER protein expression in the gastric and colonic smooth muscle strips using β-actin as a loading control. Our data shows that in both tissues and across both sexes, *db/db* mice exhibited suppressed GPER protein expression compared to the WT controls (Fig. 3). This is consistent with the mRNA data we reported above. Within the same sex, GPER protein levels are higher in the gastric and colonic smooth muscles of the WT male mice when compared to the *db/db* male mice; WT females also had higher GPER expression when compared to *db/db* mice (Fig. 3). WT female mice gastric and colonic smooth muscles had higher GPER expression when compared to WT males, and *db/db* females had higher GPER expression when compared to *db/db* males (Fig. 3).

### *GPER* downregulation in the stomach and colon of *db/db* mice is associated with promoter hypermethylation

**Figure 4:**
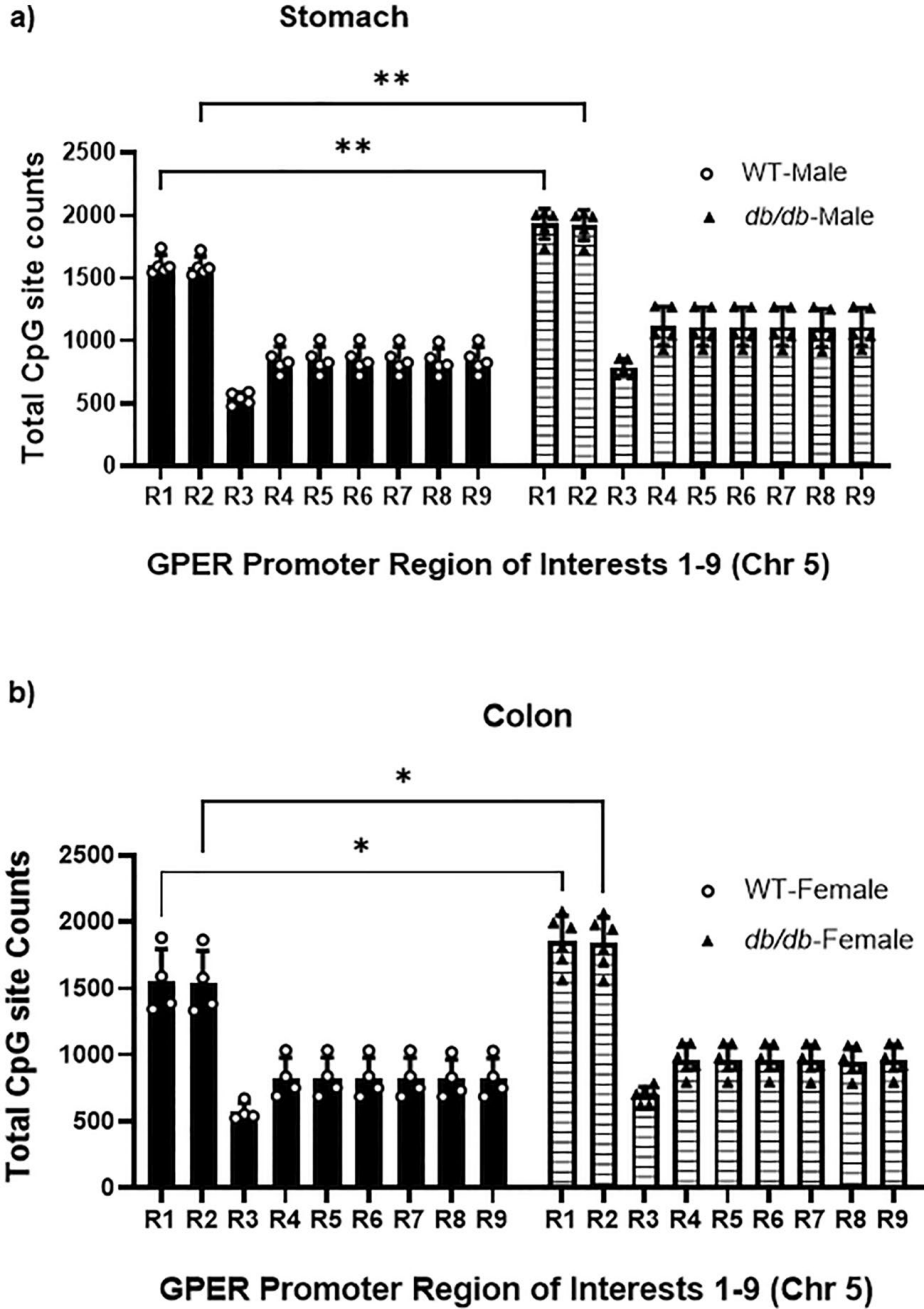
GPER promoter CpG methylation counts of the male gastric and female colonic smooth muscle. (**a**) Bisulfite DNA methylation sequencing data comparing the methylation potential of nine regions (R1-R9) within the GPER promoter sequence of the male WT versus *db/db* mouse gastric smooth muscles. The bar size represents the mean total CpG counts of n = 5 biologically independent experiments for each region. The ANOVA-generated p-value is significant at **p = 0.005 when the R1 and R2 of the groups are compared. (**b**) Bisulfite DNA methylation sequencing data comparing the methylation potential of nine regions (R1-R9) within the GPER promoter sequence of the female WT versus *db/db* mice. The bar size represents the mean CpG counts of n = 5 biologically independent female mice for each region. The ANOVA-generated p-values for the colonic smooth muscle are significant at, *p = 0.01 between the R1 and R2 of the groups.

**Figure 5:**
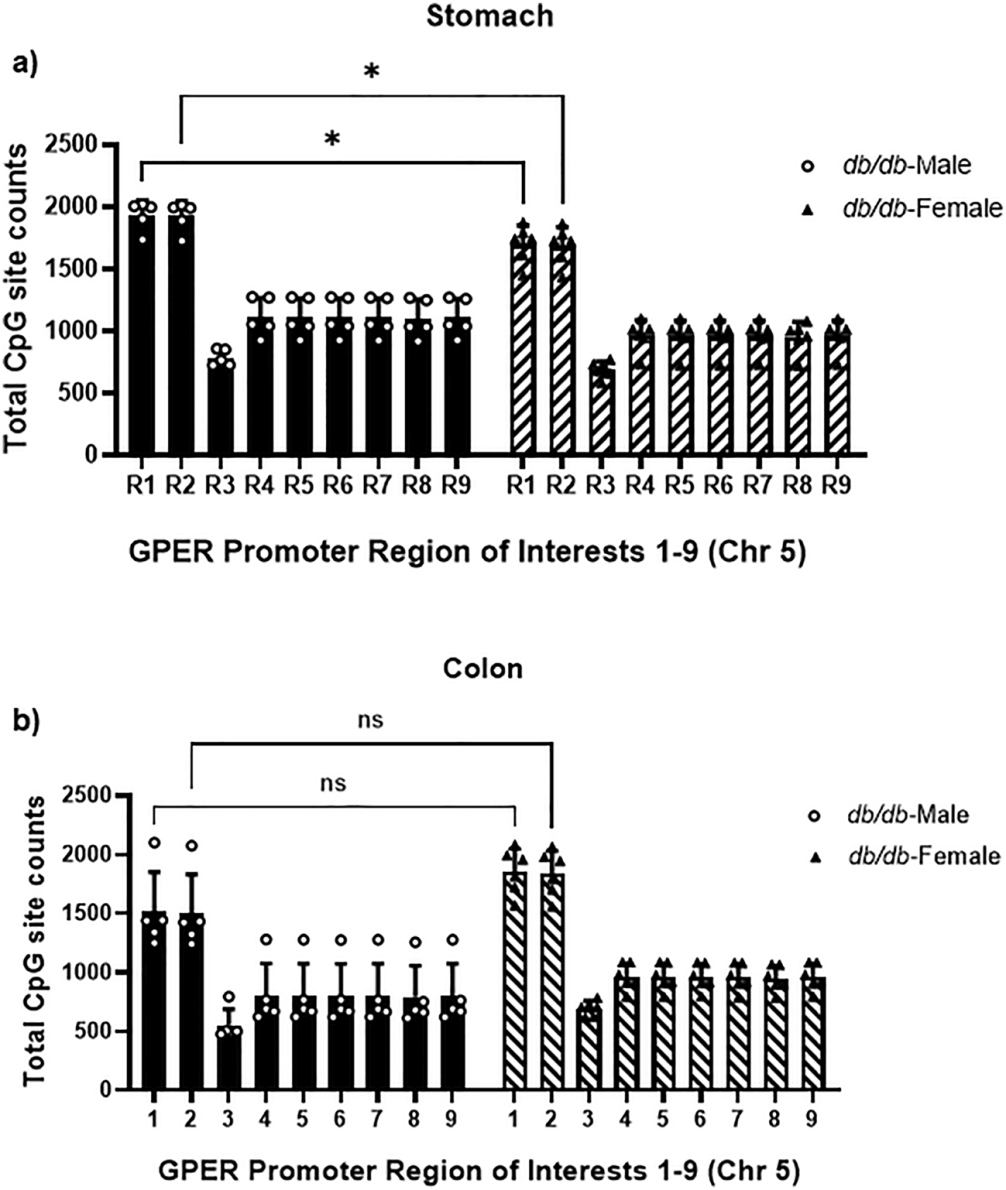
GPER promoter CpG methylation counts of the *db/db* male and *db/db* female gastric and colonic smooth muscle. (**a**) Bisulfite DNA methylation sequencing data comparing the methylation potential of nine regions (R1-R9) within the GPER promoter sequence of the *db/db* male versus *db/db* female mouse gastric smooth muscles. The bar size represents the mean total CpG counts of n = 5–6 biologically independent experiments for each region. The ANOVA-generated p-value is significant at *p = 0.003 when the R1 and R2 of the groups are compared. (**b**) Bisulfite DNA methylation sequencing data comparing the methylation potential of nine regions (R1–R9) within the GPER promoter sequence of the *db/db* male versus *db/db* female mouse colonic smooth muscles. The bar size represents the mean total CpG counts of n = 5 biologically independent experiments for each region. The ANOVA-generated p-value is not significant when the R1 and R2 of the groups are compared.

To explore the molecular mechanism of *GPER* downregulation in the gastric and colonic smooth muscles, we measured the *GPER* gene promoter methylation ratio. Nine regions were sequenced (R1-R9) within the *GPER* promoter sequence. The *GPER* promoter targeted bisulfite methylation sequencing of the WT versus *db/db* male gastric smooth muscle had significantly higher total CpG counts in the *db/db* mice compared to WT (20.8 ± 5.6% increase in region 1 and 21.2 ± 5.7% increase in region 2) (Fig. 4a). *db/db* female mice had significantly higher CpG counts in colonic smooth muscle than WT (20.2 ± 13.5% increase in region 1 and 20 ± 13.4% increase in region 2) (Fig. 4b), as shown by the regions of interest (R1 and R2). The total CpG counts of *db/db* male gastric smooth muscle were significantly higher than that of *db/db* females (12.67 ± 0.04% increase in region 1 and 13.04 ± 0.04% increase in region 2) (Fig. 5a); however, there was no such difference in colonic smooth muscle (Fig. 5b).

**Figure 6:**
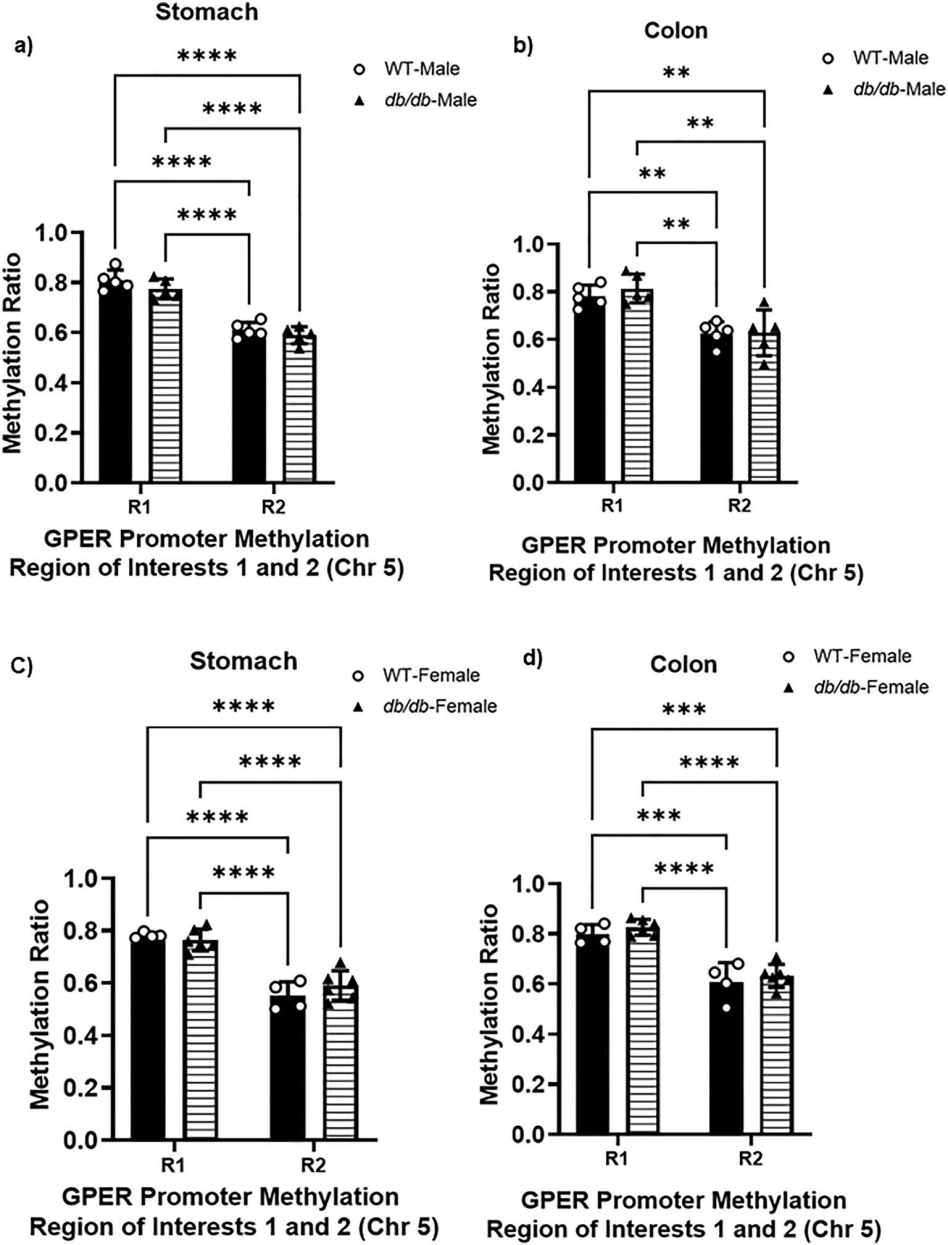
GPER promoter CpG methylation ratio of the gastric and colonic smooth muscle. (**a**) Comparison of the methylation ratio of the two regions of interest (R1 and R2) in male mouse gastric smooth muscle, calculated as the total number of methylated cytosine/total number of cytosine residues found in each region. The bar size represents the mean methylation ratio of n = 5 biologically independent mice in R1 and R2. The ANOVA-generated p-values are significant at ****p < 0.0001 both for R1 and R2. (**b**) Comparison of the methylation ratio of the two regions of interest (R1 and R2) of male mouse colonic smooth muscle, calculated as the total number of methylated cytosine/total number of cytosine residues found in each region. The bar size represents the mean methylation ratio of n = 5 biologically independent mice in R1 and R2. The ANOVA-generated p-values are significant at **p = 0.003 for R1, and ***p = 0.0008 for R2. (**c**) Comparison of the methylation ratio of the two regions of interest (R1 and R2) in female mouse gastric smooth muscle, calculated as the total number of methylated cytosine/total number of cytosine residues found in each region. The bar size represents the mean methylation ratio of n = 5 biologically independent mice in R1 and R2. The ANOVA-generated p-values are significant at ****p < 0.0001 not only for R1 and R2, but also between R1 and R2 of WT and *db/db* mice. (**d**) Comparison of the methylation ratio of the two regions of interest (R1 and R2) of female mouse colonic smooth muscle, calculated as the total number of methylated cytosine/total number of cytosine residues found in each region. The bar size represents the mean methylation ratio of n = 5 biologically independent mice in R1 and R2. The ANOVA-generated p-values are significant at ***p = 0.0002 for WT R1 vs R2 WT, and ***p = 0.0003 for R1 WT vs R2 *db/db*; at the same time, ****p < 0.0001 when comparing R1 of *db/db* vs R2 of WT mice, and ****p < 0.0001 comparing R1 of *db/db* mice vs R2 of *db/db* mice.

**Figure 7:**
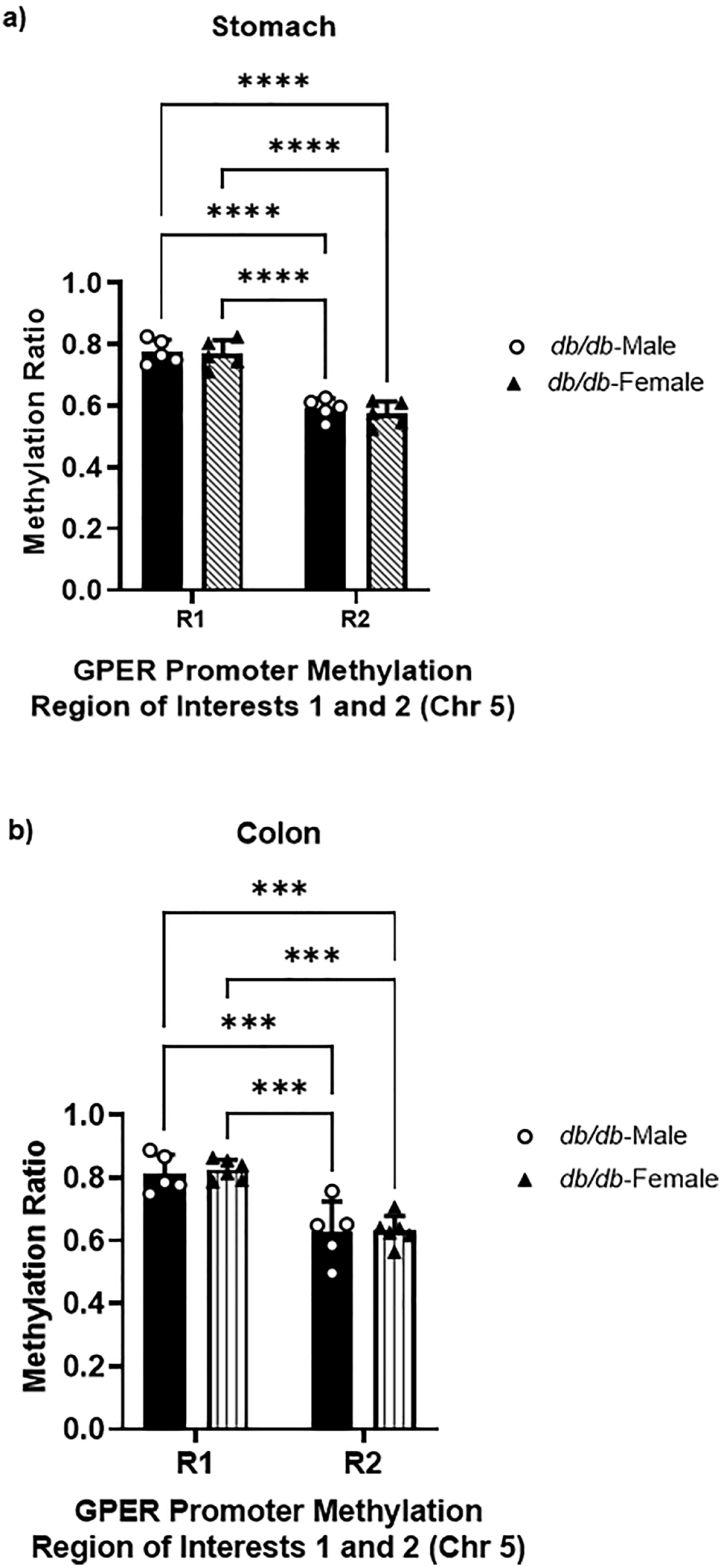
GPER promoter CpG methylation ratio of the *db/db* gastric and colonic smooth muscle. (**a**) Comparison of the methylation ratio of the two regions of interest (R1 and R2) of *db/db* male and female mouse gastric smooth muscle, calculated as the total number of methylated cytosine/total number of cytosine residues found in each region. The bar size represents the mean methylation ratio of n = 5 biologically independent mice in R1 and R2. The ANOVA-generated p-values are significant at ****p = 0.0001 for *db/db* male R1 vs R2 *db/db* male, and ****p = 0.0001 for R1 *db/db* male vs R2 *db/db* female; at the same time, ****p < 0.0001 when comparing R1 of *db/db* female vs R2 of *db/db* male mice, and ****p < 0.0001 comparing R1 of *db/db* female mice vs R2 of *db/db* female mice. (**b**) Comparison of the methylation ratio of the two regions of interest (R1 and R2) of *db/db* male and female mouse colonic smooth muscle, calculated as the total number of methylated cytosine/total number of cytosine residues found in each region. The bar size represents the mean methylation ratio of n = 5 biologically independent mice in R1 and R2. The ANOVA-generated p-values are significant at ***p = 0.0003 for *db/db* male R1 vs R2 *db/db* male, and ***p = 0.0004 for R1 *db/db* male vs R2 *db/db* female; at the same time, ****p < 0.0002 when comparing R1 of *db/db* female vs R2 of *db/db* male mice, and ****p < 0.0003 comparing R1 of *db/db* female mice vs R2 of *db/db* female mice.

However, the methylation ratio seems higher in R1 compared to R2 across male and female WT and *db/db* in both tissues: 24.5 ± 1.1% higher in male WT gastric smooth muscle in region 1 compared to region 2 (Fig. 6a). It is 23.9 ± 1.5% higher in male *db/db* gastric smooth muscle in region 1 compared to region 2 and 19.9 ± 3.3% higher in male WT colonic smooth muscle in region 1 compared to region 2. Moreover, it is 29.9 ± 4.4% higher in male *db/db* colonic smooth muscle in region 1 compared to region 2 (Fig. 6b). It is 29.5 ± 2.7% higher in female WT gastric smooth muscle in region 1 compared to region 2 and 35.4 ± 2% higher in female *db/db* gastric smooth muscle in region 1 compared to region 2. Moreover it is 23.9 ± 3.3% higher in female WT colonic smooth muscle in region 1 compared to region 2 and 32.3 ± 2.7% higher in female *db/db* colonic smooth muscle in region 1 compared to region 2 (Fig. 6c and d). It is 23.9 ± 1.5% higher in *db/db* male gastric smooth muscle in region 1 compared to region 2 and 31.9 ± 2% higher in female *db/db* gastric smooth muscle in region 1 compared to region 2 (Fig. 7a). Moreover, it is 23.1 ± 3.5% higher in male *db/db* colonic smooth muscle in region 1 compared to region 2 and 25.4 ± 0.6% higher in female *db/db* colonic smooth muscle in region 1 compared to region 2 (Fig. 7b).

### *GPER* promoter hypermethylation in the stomach and colon of *db/db* mice is associated with the upregulation of DNMTs

**Figure 8:**
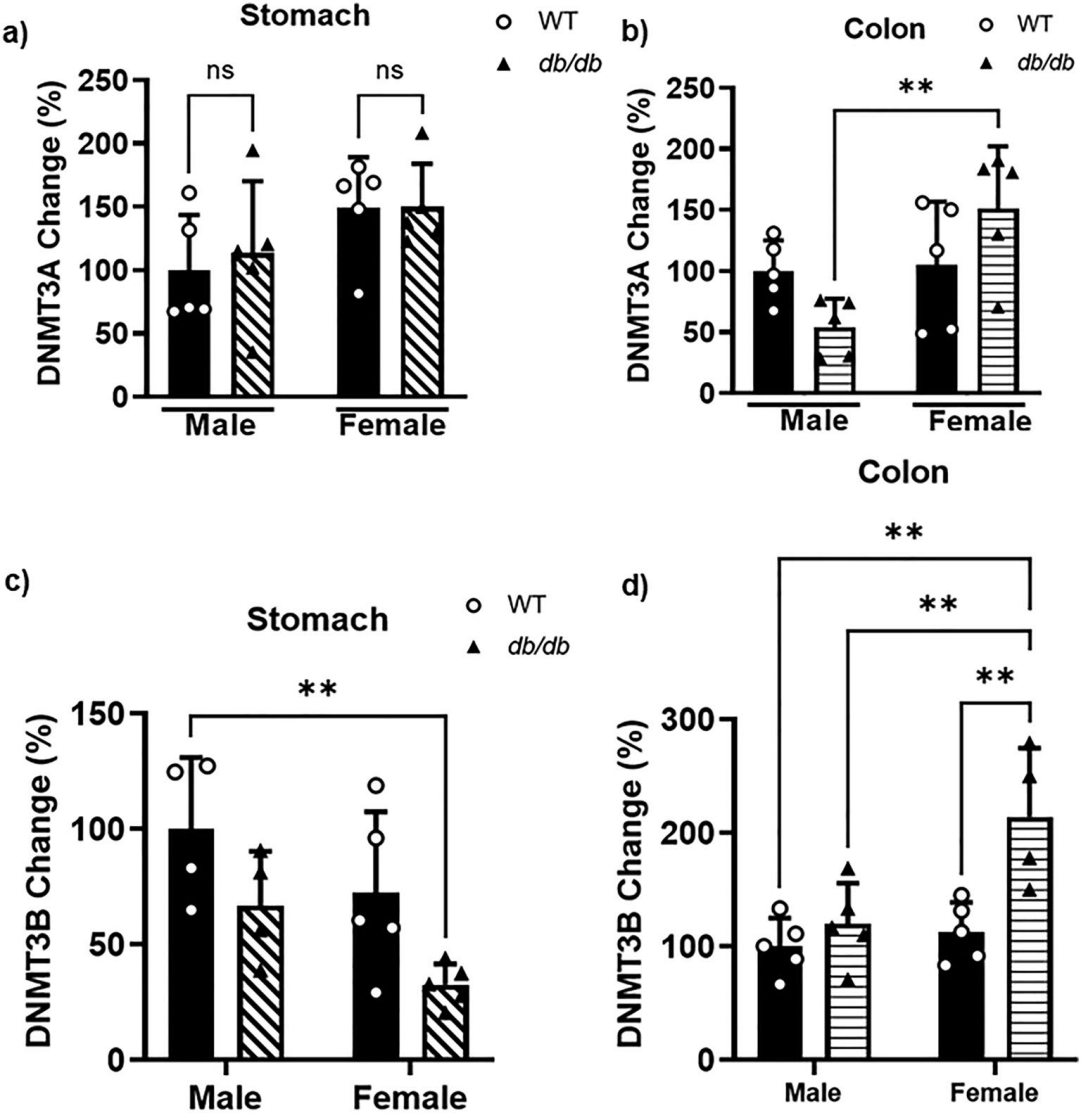
Gastric and colonic content of DNMT3A and DNMT3B. (**a**) ELISA results comparing the protein levels of DNMT3A in the gastric smooth muscle of male versus female WT versus *db/db* mice. The bar size represents the mean DNMT3A levels of n = 4–5 biologically independent mice. The ANOVA-generated p-value is not significant at p > 0.05. (**b**) ELISA results comparing the protein levels of DNMT3A in the colonic smooth muscle of male versus female WT versus *db/db* mice. The bar size represents the mean DNMT3A levels of n = 4–5 biologically independent mice. The ANOVA-generated p-value is significant at **p = 0.007. (**c**) ELISA results comparing the protein levels of DNMT3B in the gastric smooth muscle of male versus female WT versus *db/db* mice. The bar size represents the mean DNMT3B levels of n = 4–5 biologically independent mice. The ANOVA-generated p-value is significant at **p = 0.009. (**d**) ELISA results comparing the protein levels of DNMT3B in the colonic smooth muscle of male versus female WT versus *db/db* mice. The bar size represents the mean DNMT3B levels of n = 4–5 biologically independent mice. The ANOVA-generated p-value is significant at **p = 0.005 when comparing any of the groups and sexes to *db/db* female mice.

We evaluated DNMT3A and DNMT3B levels in both male and female WT versus *db/db* gastric and colonic smooth muscle to further explain methylation differences. No significant change in DNMT3A was observed in *db/db* male and female gastric smooth muscle relative to WT (Fig. 8a). Higher levels of DNMT3A were found in female *db/db* mice colonic smooth muscle compared to their male counterparts (291 ± 92% higher) but not between male and female WT mice (Fig. 8b). No significant change in DNMT3B was observed between *db/db* male or female gastric smooth muscles, nor between WT male and female mice; however, WT male gastric smooth muscle had 66.46 ± 8.6% higher DNMT3B levels (Fig. 8c). In the colonic smooth muscle, higher levels of DNMT3B were found in the *db/db* female mice, which were significantly higher than the WT male (by 103.6 ± 13.9%), *db/db* male (by 81.6 ± 11.6%) and WT female (by 87.9 ± 28.24%) (Fig. 8d). Moreover, *db/db* females had significantly lower gastric smooth muscle DNMT3B levels than WT males and significantly higher colonic smooth muscle DNMT3B levels than WT and *db/db* males (Fig. 8c and d).

### *GPER* downregulation in the stomach and colon of *db/db* mice is associated with a decreased relative abundance of H3K4me3 and H3K27ac marks

**Figure 9:**
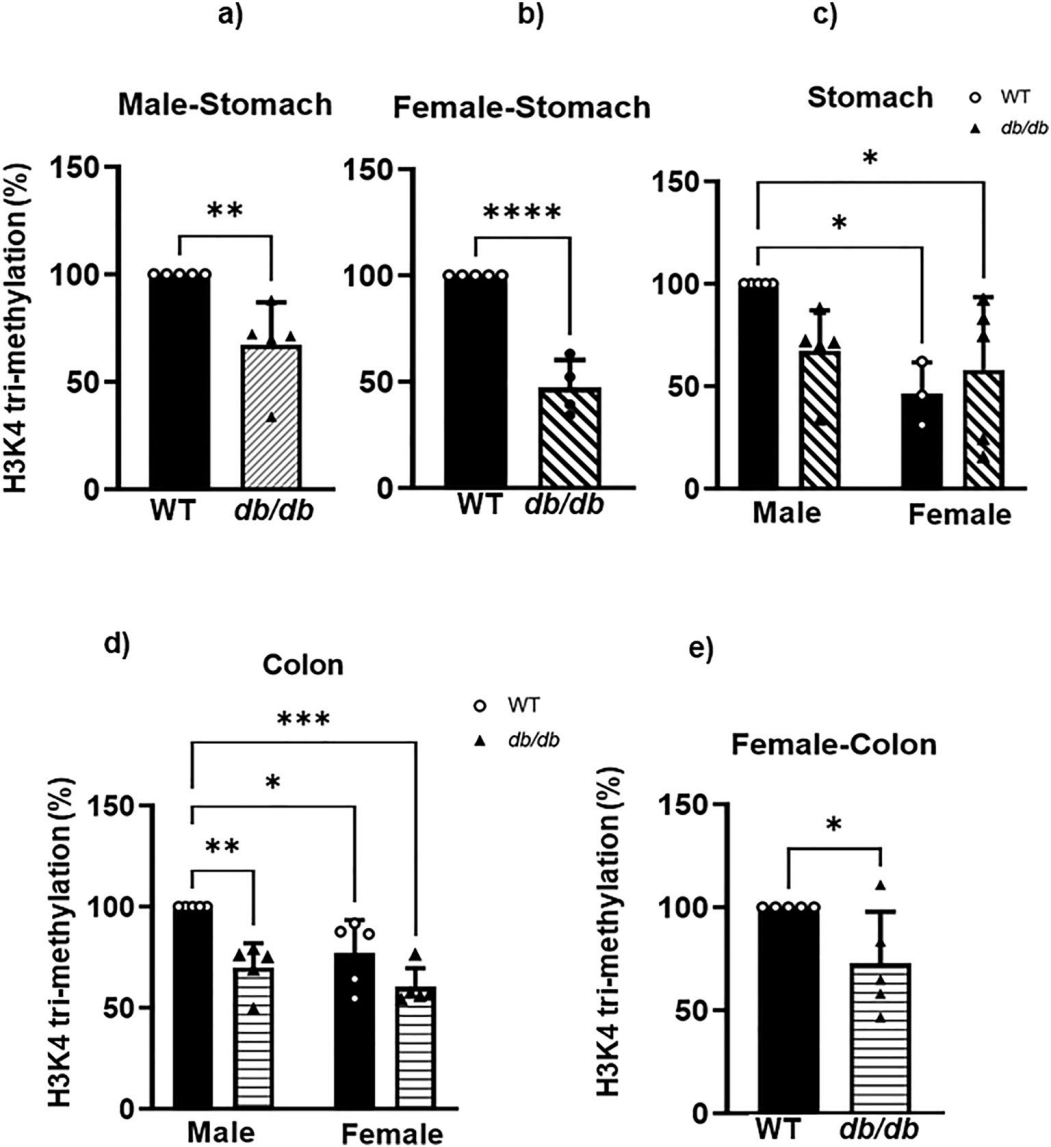
Gastric and colonic content of H3K4me3 marked chromatin. ELISA results comparing the proportion of chromatin marked by H3K4me3 in the gastric and colonic smooth muscle of male versus female WT versus *db/db* mice. The bar size represents the mean H3K4me3 levels of n = 5 biologically independent mice per group. (**a**) The t-test-generated p-values are significant at **p = 0.001 (WT vs *db/db* male). (**b**) The t-test-generated p-values are significant at ****p < 0.0001 (WT female vs *db/db* female). (**c**) The ANOVA-generated p-values are significant at *p = 0.02 (WT male vs WT female), and *p = 0.04 (WT male vs *db/db* female). (**d**) The ANOVA-generated p-value is significant at **p = 0.003 (WT male vs *db/db* male), *p = 0.02 (WT male vs WT female), ***p = 0.0002 (WT male vs *db/db* female) (**e**) The t-test-generated p-value is significant at *p = 0.04 (WT female vs *db/db* female).

To explore whether histone modification could also play a role in *GPER* downregulation in addition to DNA hypermethylation, we extracted the total histones from both muscle strips. We used the EpiQuik H3K4me3 and H3K27ac ELISA assay kits to check the relative abundance of the aforementioned histone marks. We found that the reduction of the H3K4me3 mark in the *db/db* male mice compared to the WT was statistically significant in the gastric and colonic smooth muscles. In the male gastric smooth muscle of *db/db*, the H3K4me3 mark was 33 ± 8.9% lower when compared to the WT control (Fig. 9a). In the female gastric smooth muscle of *db/db*, the H3K4me3 mark was 53 ± 5.8% lower when compared to the WT control (Fig. 9b). In the female gastric smooth muscle of WT, the H3K4me3 mark was 54 ± 7.0% lower when compared to the WT males. When compared to the female gastric smooth muscle of *db/db*, the H3K4me3 mark decrease was 42 ± 16% (Fig. 9c). In the male colonic smooth muscle of *db/db*, the H3K4me3 mark was 30 ± 5.3% lower when compared to the WT control (Fig. 9d). This difference was 23 ± 7.3% comparing WT male mice against female WT mice, and 40 ± 4% against *db/db* female mice (Fig. 9d). In the female colonic smooth muscle of *db/db*, the H3K4me3 mark was 37 ± 7% lower when compared to the WT control (Fig. 9e). All the above-mentioned differences were statistically significant. No significant differences in H3K4me3 were found, however, between the *db/db* male and female gastric or colonic smooth muscles (Fig. 9c and d).

**Figure 10:**
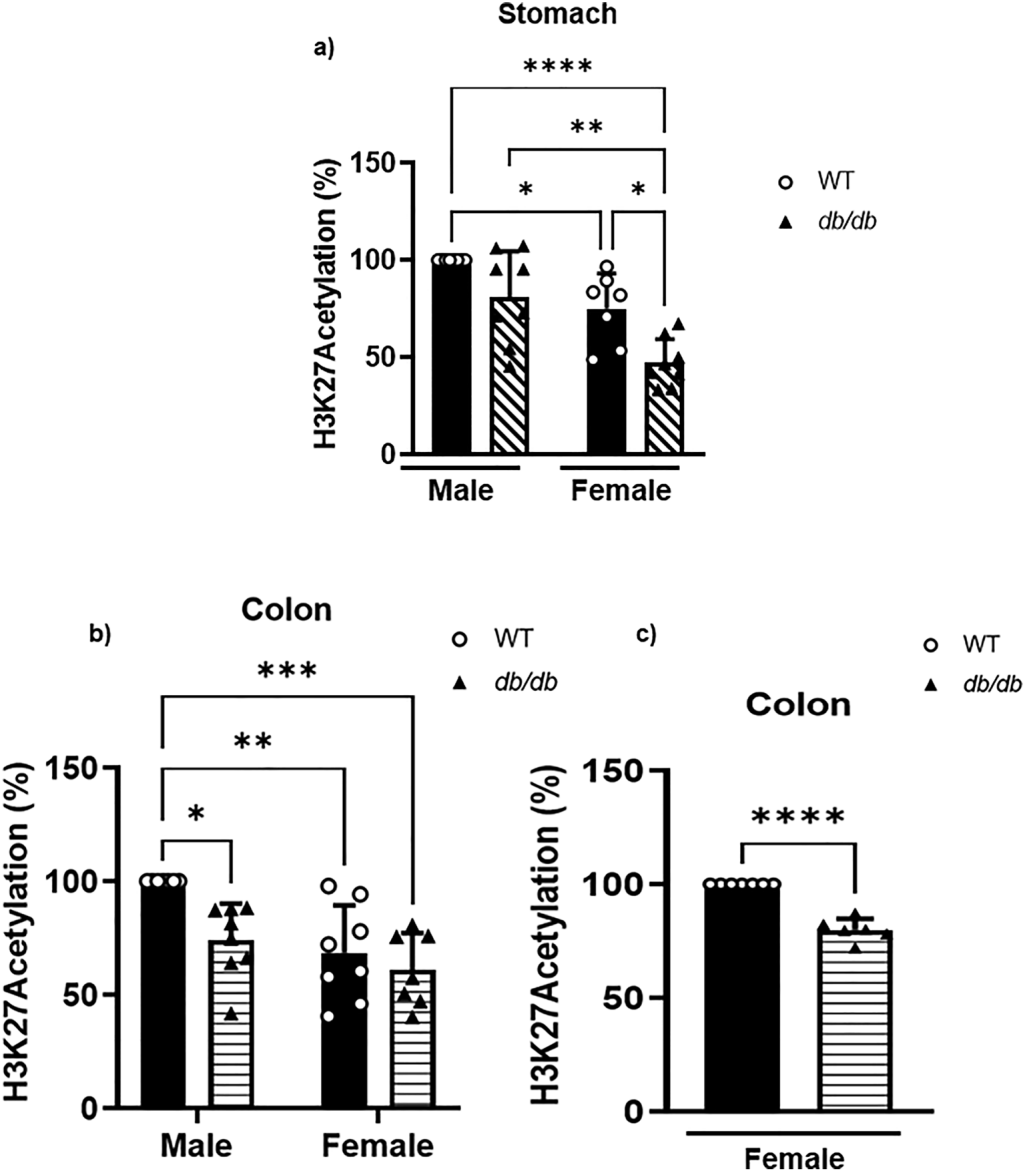
Gastric and colonic content of H3K27ac marked chromatin. (**a**) ELISA results comparing the proportion of chromatin marked by H3K27ac in the gastric smooth muscle of male versus female WT versus *db/db* mice. The bar size represents the mean H3K27ac levels of n = 5 biologically independent mice per group. The ANOVA-generated p-values are significant at *p = 0.02 (WT male vs WT female), ****p < 0.0001 (WT male vs *db/db* female), **p = 0.001 (*db/db* male vs *db/db* female), and *p = 0.01 (WT female vs *db/db* female). (**b**) ELISA results comparing the proportion of chromatin marked by H3K27ac in the colonic smooth muscle of male versus female WT versus *db/db* mice. The bar size represents the mean H3K27ac levels of n = 5 biologically independent mice. The ANOVA-generated p-value is significant at *p = 0.02 (WT male vs *db/db* male), and **p = 0.005 for WT male vs WT female, and **p = 0.002 for WT male vs *db/db* female. (**c**) ELISA results comparing the proportion of chromatin marked by H3K27ac in the colonic smooth muscle of WT versus *db/db* female mice. The bar size represents the mean H3K27ac levels of n = 5 biologically independent mice. The t-test-generated p-value is significant at ****p < 0.00001.

We found that the reduction of the H3K27ac mark in the *db/db* male mice compared to the WT was statistically significant in the colonic smooth muscles (Fig. 10). In the female gastric smooth muscle of WT, the H3K27ac mark was 25 ± 6.8% lower when compared to the WT males (Fig. 10a). When comparing to the female gastric smooth muscle of *db/db* to male gastric smooth muscle of *db/db*, the H3K27ac mark decrease was 35 ± 6.3% was lower in females (Fig. 10a). In the female gastric smooth muscle of WT, the H3K27ac mark was 32 ± 0.1% lower when compared to the WT female (Fig. 10a). When comparing to the female smooth muscle of *db/db* to male gastric smooth muscle of WT, the H3K27ac mark decrease was 51.1 ± 4.9% was lower in females. In the male colonic smooth muscle of *db/db*, the H3K27ac mark was 26 ± 5.7% lower when compared to the WT control; but no significant difference was found between the H3K27ac of the colonic smooth muscles of *db/db* male and female mice (Fig. 10b). This difference was 32 ± 7.4% comparing WT male mice against female WT mice, and 47 ± 10% against *db/db* female mice (Fig. 10b). In the female colonic smooth muscle of *db/db*, the H3K27ac mark was 20 ± 2% lower when compared to the WT control (Fig. 10c). All the above-mentioned differences were statistically significant.

### The decreased H3K4me3 and H3K27ac marks in the *db/db* mice led to their reduced enrichment around the *GPER* promoter

**Figure 11:**
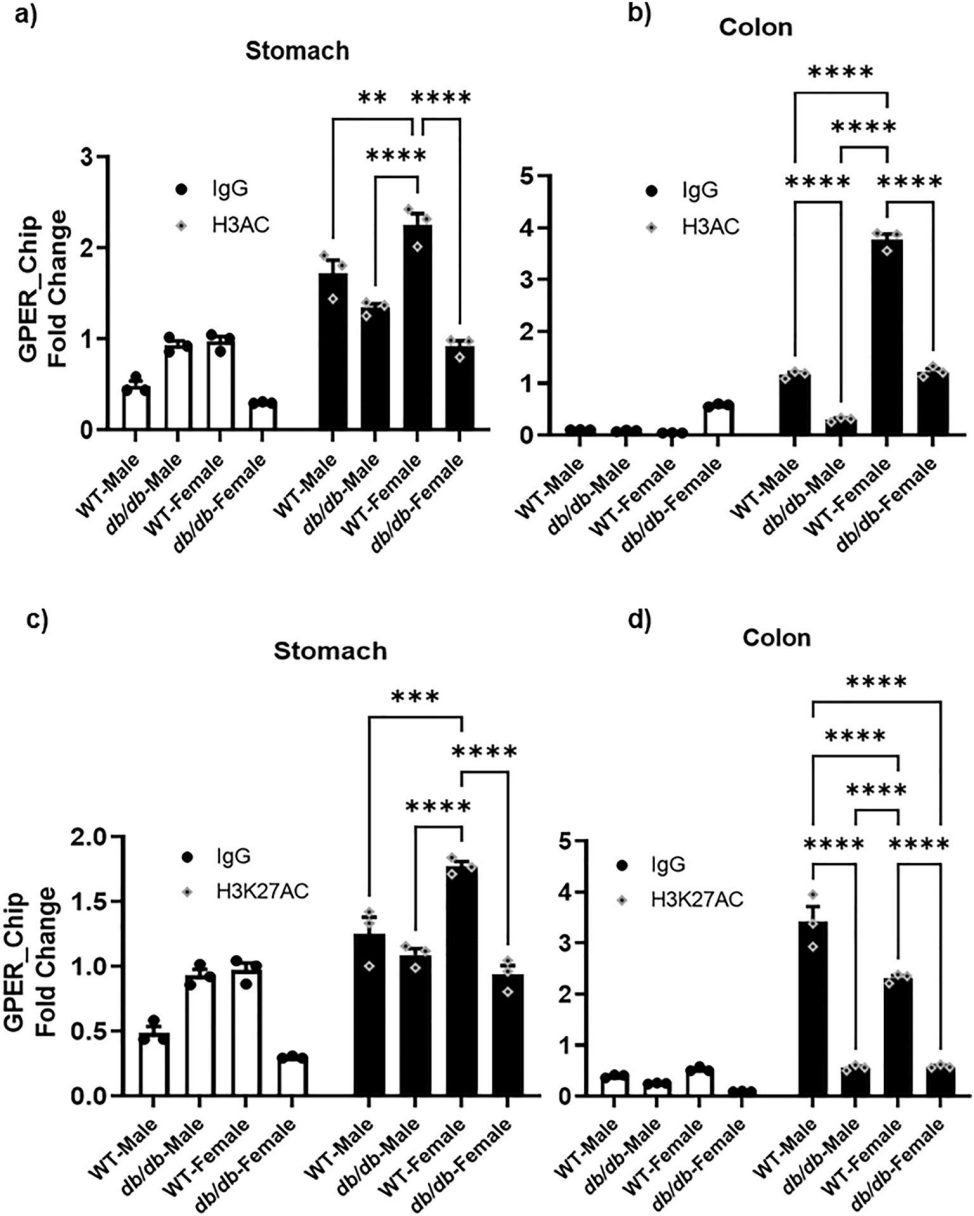
Gastric and colonic enrichment of global H3ac and H3K27ac marks around GPER promoter. (**a**) ChIP assay results comparing the enrichment of global H3ac (black bars) compared to IgG (white bars) in the gastric smooth muscle of male versus female WT versus *db/db* mice. The bar size represents the mean global H3ac levels of n = 3 biologically independent mice. The ANOVA-generated p-values for H3AC are significant at **p = 0.008 (WT male vs WT female), ****p < 0.0001 (*db/db* male vs WT female), ****p < 0.0001 (WT female vs *db/db* female). (**b**) ChIP assay results comparing the enrichment of global H3ac (black bars) compared to IgG (white bars) in the colon of male versus female WT versus *db/db* mice. The bar size represents the mean global H3ac levels of n = 3 biologically independent mice. The ANOVA-generated p-values are significant at ****p < 0.0001 for the following comparisons: WT male vs *db/db* male, WT male vs WT female, *db/db* male vs WT female, WT female vs *db/db* female mice. (**c**) ChIP assay results comparing the enrichment of H3K27ac (black bars) compared to IgG (white bars) in the gastric smooth muscle of male versus female WT versus *db/db* mice. The bar size represents the mean H3K27ac levels of n = 3 biologically independent mice. The ANOVA-generated p-values are significant at ***p = 0.0001 (WT male vs WT female), and ****p < 0.0001 for *db/db* male vs WT female and WT female vs *db/db* female. (**d**) ChIP assay results comparing the enrichment of H3K27ac (black bars) compared to IgG (white bars) in the colonic smooth muscle of male versus female WT versus *db/db* mice. The bar size represents the mean H3K27ac levels of n = 3 biologically independent mice. The ANOVA-generated p-values are significant at ****p < 0.0001 for the following comparisons: WT male vs *db/db* male, WT male vs WT female, WT male vs *db/db* female, *db/db* male vs WT female, and WT female vs *db/db* female.

We conducted a ChIP assay using the extracted total histone proteins to check the enrichment of our targeted histone marks around the *GPER* promoter. Interestingly, we observed a significant reduction in the enrichment of the global H3ac mark in the chromatin region harboring the *GPER* promoter of both the male and female *db/db* mice in both gastric and colonic smooth muscle tissues (Fig. 11a and b), except that reduction is statistically insignificant in the stomach of male WT versus *db/db* mice (Fig. 11a). Males generally experienced a more significant reduction in the enrichment of this mark across the WT and *db/db* groups in both tissues except for the slight but statistically insignificant increase in the WT versus *db/db* mice in the male stomach (Fig. 11a).

Similar to the global H3ac, we observed a significantly lower enrichment of the more specific histone acetylation mark (H3K27ac), in the *db/db* mice in both tissues (Fig. 11c and d), and consistent with the global H3ac data, the reduction was statistically insignificant in the *db/db* versus WT males in the stomach (Fig. 11a). Comparing different genders, we found that the female WT mice had more significant enrichment of the mark than their male counterparts in the stomach, but a slight (though insignificant) increase in the *db/db* male versus female observed in the same tissue (Fig. 11c). In the colon; however, a significant increase in the mark was observed in the male versus female WT mice. Still, no significant change was seen in the gastric or colonic smooth muscles of *db/db* males versus females H3ac nor H3K27ac (Fig. 11).

**Figure 12:**
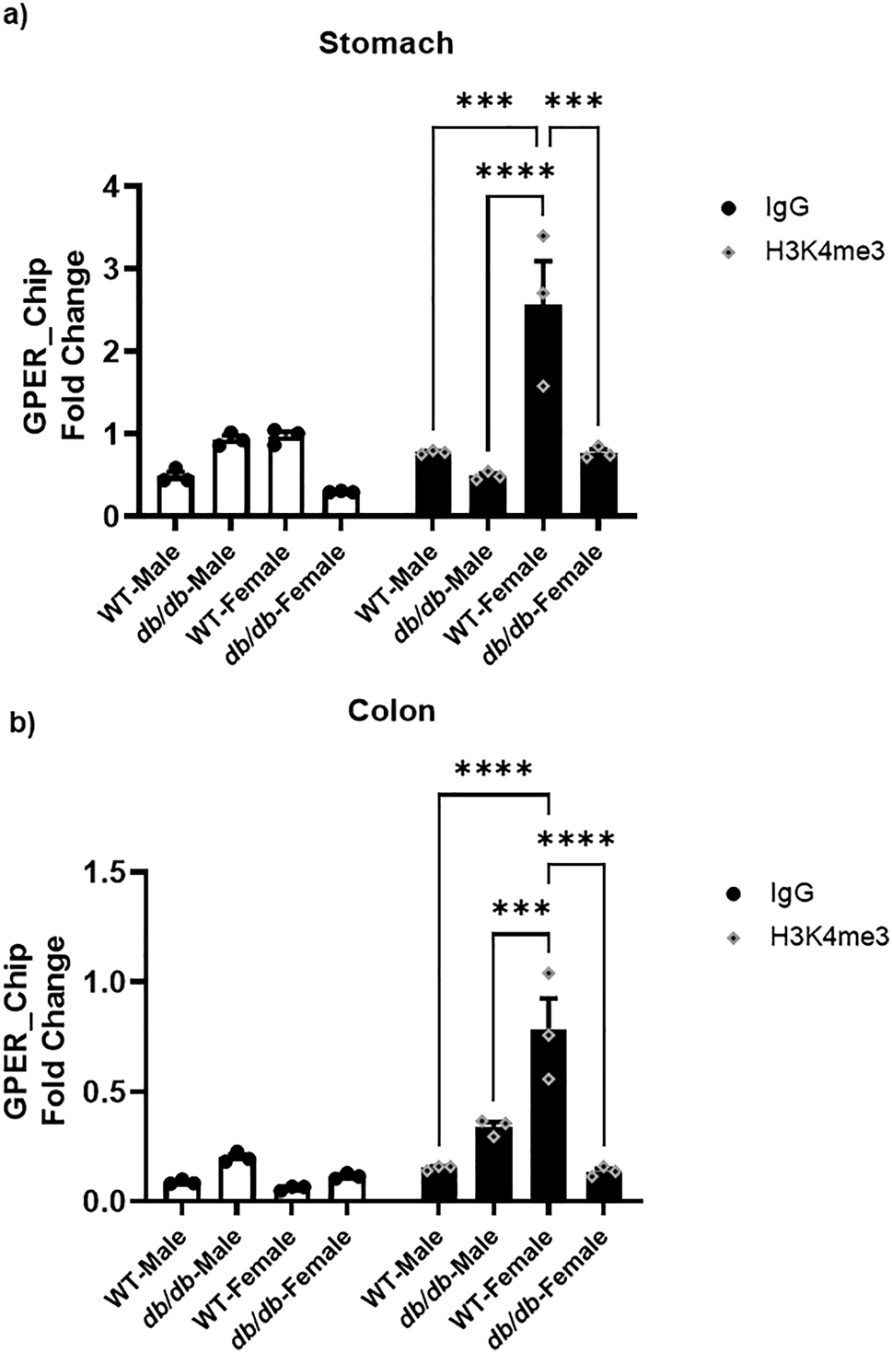
Gastric and colonic enrichment of H3K4me3 marks around GPER promoter. (**a**) ChIP assay results comparing the enrichment of H3K4me3 (black bars) compared to IgG (white bars) in the gastric smooth muscle of male versus female WT versus *db/db* mice. The bar size represents the mean H3K4me3 levels of n = 3 biologically independent mice. The ANOVA-generated p-values are significant at ***p = 0.0001 (WT male vs WT female, and WT female vs *db/db* female), and ****p < 0.0001 (*db/db* male vs WT female). (**b**) ChIP assay results comparing the enrichment of H3K4me3 (black bars) compared to IgG (white bars) in the colonic smooth muscle of male versus female WT versus *db/db* mice. The bar size represents the mean H3K4me3 levels of n = 3 biologically independent mice. The ANOVA-generated p-values are significant at ***p < 0.001 (*db/db* male vs WT female), and ****p < 0.0001 (WT male vs WT female and WT female vs *db/db* female).

For the H3K4me3 mark, only the female *db/db* mice exhibited a statistically significant reduction in the enrichment of the mark compared to their WT controls in both tissues (Fig. 12a and b). Comparing the genders, we observed that the female WT mice had more significant enrichment of the mark than the males in both tissues. However, no significant change was seen in the gastric or colonic smooth muscles of *db/db* males versus females H3K4me3 (Fig. 12a and b).

### GPER deficiency caused variations in daily fecal pellet production patterns of mice

**Figure 13:**
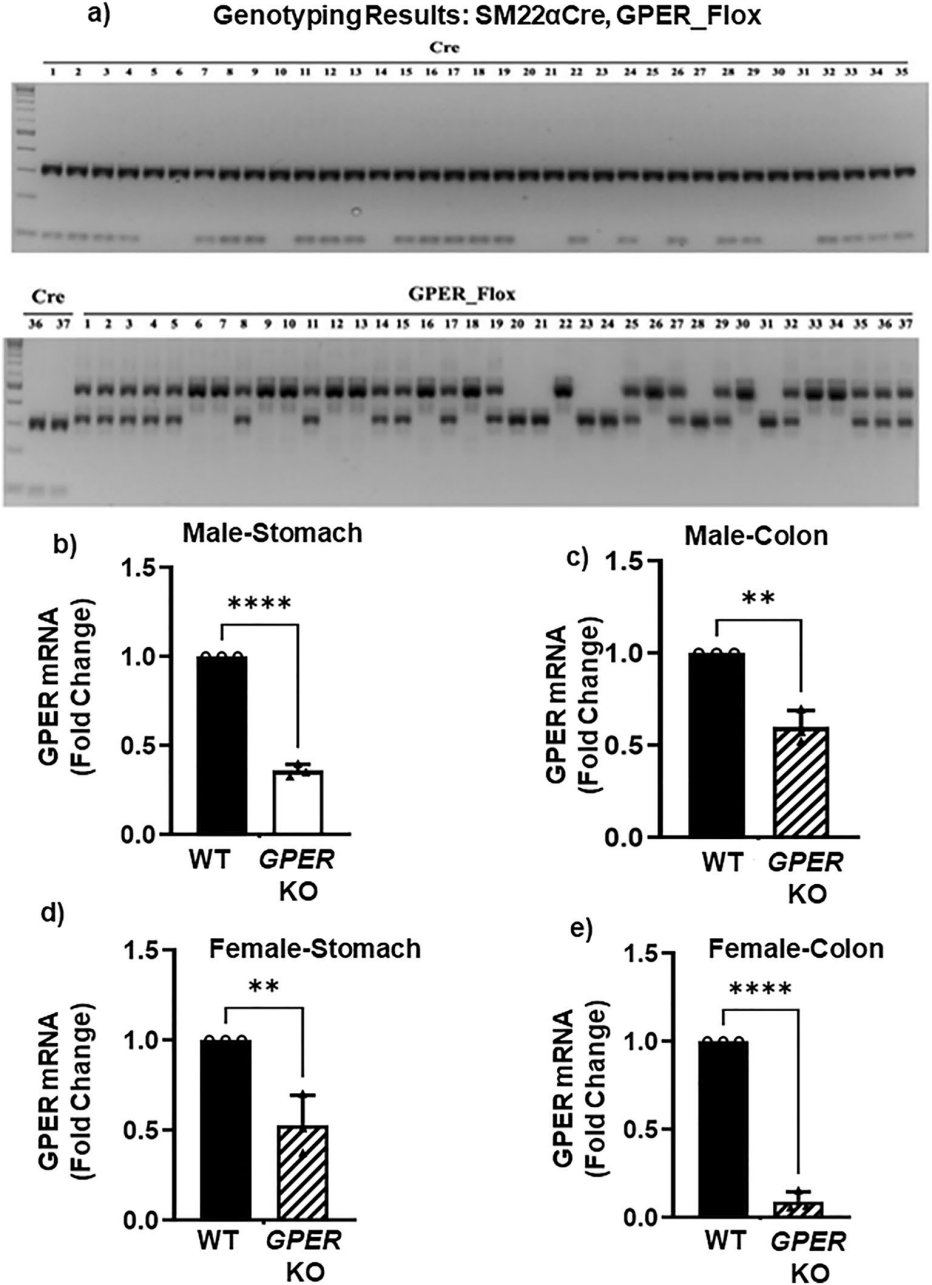
Smooth muscle specific *GPER* KO genotype results. (**a**) (top): PCR results of experimental mice probing for the Cre gene. The Cre gene was found in mouse samples 1, 2, 3, 4, 7, 8, 9, 11, 12, 13, 15, 16, 17, 18, 19, 22, 24, 26, 28, 29, 32, 33, 34, 35. (Bottom). (Left) continuation of the results for the Cre gene, positive for samples 36 and 37. This is followed by the PCR results of experimental mice probing for the GPER Flox gene. The gene was found in the mice, 1–5, 8, 11, 14, 15, 17, 19, 25, 27, 29, 32, 35–37 (heterozygous), 6, 7, 9, 10, 12, 13, 16, 18, 22, 26, 30, 33 and 34 (homozygous). The floxed gene was not found in the following samples: 20, 21, 23, 24, 28, 31. Overall, the mice with the GPER Flox/Flox (homozygous) X Cre combination correspond to samples 7, 9, 12, 13, 16, 18, 22, 26, 33, 34, that is, smooth muscle-specific* GPER* knockout (KO) mice. Tagln_Cre: oIMR1084/1085/7338/7339 (WT: 324 bp; Cre: 100 bp) GPER_Flox 4G2/4G5 (WT:350 bp; Flox:530 bp). (**b**) qRT-PCR analysis showing *GPER* mRNA expression (in fold change) of the WT male mice (black bar) versus smooth muscle specific *GPER* KO male mice (gray bar) in gastric smooth muscle. The bar size represents the mean of n = 2–3 WT and smooth muscle specific *GPER* KO animals per group. Results were deemed significant when p < 0.05. The t-test generated p-value is significant, at ***p = 0.0001 (**c**) *GPER* mRNA expression (in fold change) of the WT male (black bar) versus smooth muscle specific* GPER* KO (gray bar) male mice in colonic smooth muscle. The bar size represents the mean of n = 2–3 biologically independent WT and smooth muscle specific *GPER* KO animals per group. The t-test generated p-value is significant, at *p = 0.0276. (**d**)* GPER* mRNA expression (in fold change) of the WT female (black bar) versus smooth muscle specific *GPER* KO female (gray bar) mice in gastric smooth muscle. The bar size represents the mean of n = 2–3 biologically independent WT and smooth muscle specific *GPER* KO animals per group. The t-test generated p-value is significant, at *p = 0.0221. (**e**) *GPER* mRNA expression (in fold change) of the WT female (black bar) versus smooth muscle specific *GPER* KO female (gray bar) mice in colonic smooth muscle. The bar size represents the mean of n = 2–3 biologically independent WT and smooth muscle specific *GPER* KO animals per group. The t-test generated p-value is significant at ***p < 0.0002.

**Figure 14:**
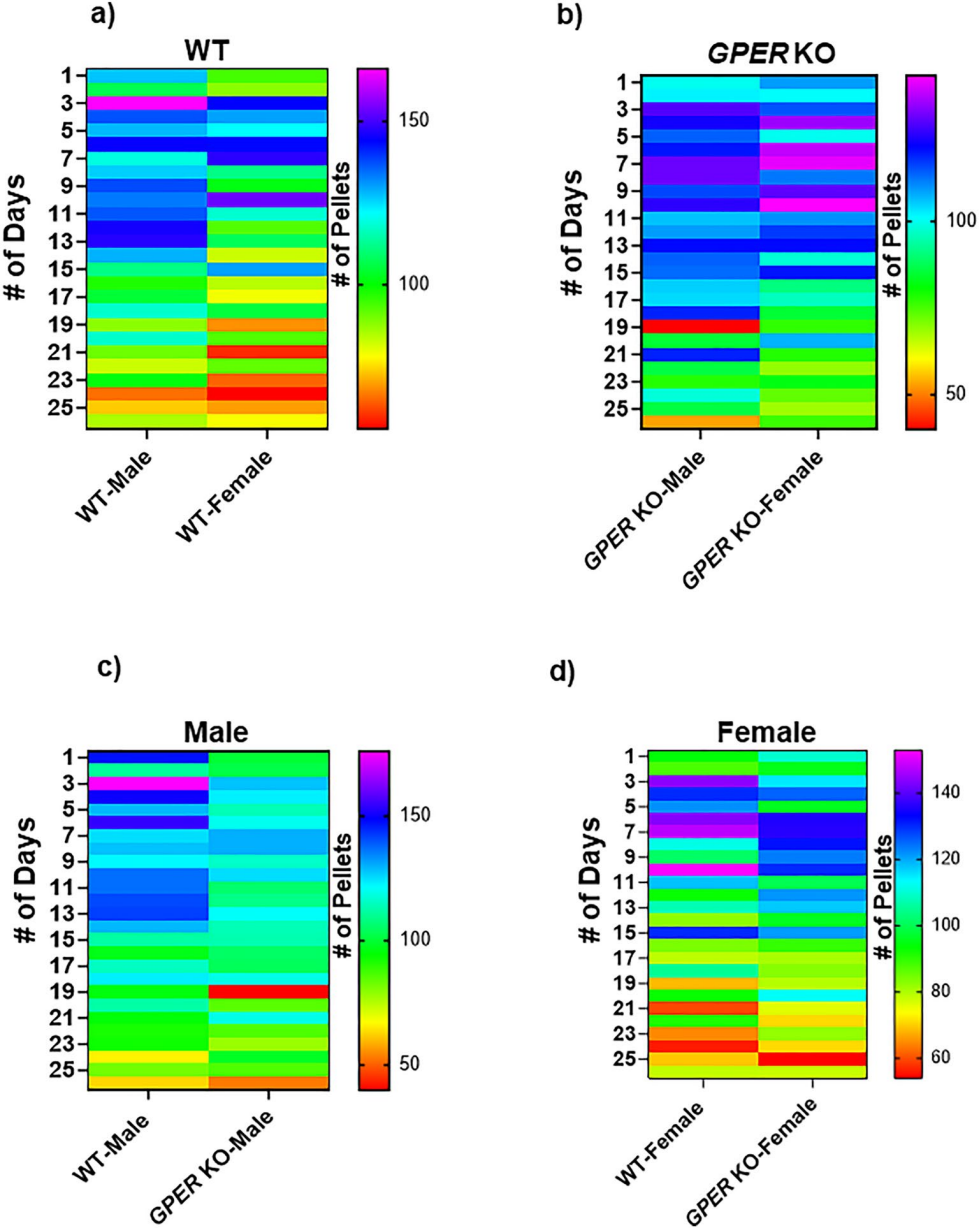
Quantification of Fecal Output in Male and Female WT and Smooth Muscle-Specific *GPER* Knockout (*GPER* KO) Mice. Fecal pellet production was assessed over a continuous 24-h period for 30 consecutive days in each mouse. The average number of fecal pellets for the 30-day duration was calculated based on data collected from three mice in each group. Results are presented as mean ± SEM for n = 3 animals in each group, with statistical significance determined at the p < 0.0001 level.

To determine whether the plausible mechanism for gastrointestinal dysmotility in diabetes may be related to GPER expression levels, our laboratory developed smooth muscle specific *GPER *KO mice by producing Tagln-Cre (also known as SM22α-Cre) x GPER flox/flox mice (Fig. 13a). Their *GPER* mRNA expression in the gastric and colonic smooth muscle was, as expected, significantly lower than that of their WT counterparts (Fig. 13b–e). There were differences in daily fecal pellet production patterns between both male and female WT and smooth muscle-specific *GPER* KO mice (Fig. 14a–d). The female pattern of daily fecal pellet production led to notable daily differences in the number of pellets produced daily by females of the same group (Fig. 14b). Meanwhile, WT and smooth muscle-specific *GPER* KO male mice showed smaller day to day variations in pellet production (Fig. 14c). However, female smooth muscle-specific *GPER* KO mice also had smaller day to day variations when compared to their WT counterparts, following a pattern more similar to the smooth muscle-specific *GPER* KO male than to the WT female (Fig. 14b and d).

## Discussion

GPER expression and activation in normal conditions is greater in the female^34–40^. Moreover, postprandial hyperglycemia slows down gastric emptying in both diabetes and in healthy individuals, which may make the effects of GPER deficiency more acute, if involved in gastroparesis^14^^,^^41^.

However, this must not lead us to assume that reductions in the expression of GPER should affect males and females equally or proportionally to the differences in GPER expression. This is because the different parts of the GI tract of males and females operate under different conditions. For instance, Gangula et al.^11^ found that the stomachs of female diabetic and healthy rats produced higher amounts of nitric oxide than those of male diabetic and healthy rats, correspondingly, even when diabetic female rats produced less nitric oxide than healthy female rats. This affects the degree of impact that further differences in the conditions may have on function.

In our case, the female mouse and human stomachs are known to empty more slowly under physiological and diabetic conditions than those of males. Considering that the baseline conditions are already unequal, it is understandable that the final conditions may not correlate proportionally under the same experimental conditions.

Firstly, we observed that females generally exhibited higher *GPER* gene and protein expression in the WT mice than males. Most importantly, we found for the first time that the *GPER* gene and protein expression is downregulated in the gastric and colonic smooth muscles of *db/db* mice compared to the WT control, with the male mice showing a more significant *GPER* suppression than females in both tissues. Interestingly, we further unveiled that the observed *GPER* downregulation is mechanistically associated with promoter hypermethylation and reduced enrichment of H3K4me3 and H3K27ac in the chromatin region around the *GPER* gene promoter. Moreover, a similar epigenetic mechanism of *GPER* downregulation was observed in the male WT mice compared to females. Therefore, considering the potential role of *GPER* signaling in maintaining normal vascular tone as well as its emerging role in regulating GIM, we conclude that type 2 diabetes (T2D) may play an essential role in *GPER* downregulation in the gut, which may lead to compromised GIM and increased burden of GIM-related diabetic complications. Furthermore, although the epidemiological data is not well established, the differences in GPER expression we observed between males and females in the WT mice may be associated with gender disparities in the prevalence and burden of diabetic-induced GIT complications with epigenetic modifications playing a pivotal role.

To the best of our knowledge, the principal findings of this study demonstrated for the first time that T2D causes downregulation of GPER expression in the gastric and colonic smooth muscles in a sex-specific manner, and epigenetic mechanisms are involved in the suppression. Our investigation, therefore, could aid in understanding the molecular basis of gender disparities in the burden of T2D complications, particularly those related to GIT dysfunction.

We first observed an overall higher *GPER* mRNA and protein expression in females than their male counterparts across both the WT and *db/db* mice's gastric and colonic smooth muscle tissues. This means that *GPER* transcription is normally more upregulated in the stomach and colon of females than in males under physiological and diabetic conditions. Higher *GPER* mRNA and protein expression in females compared to males have also been previously reported in other rodent tissues such as the pancreatic islets^34^, renal^35,36^, brain trigeminal ganglion^37^, hypothalamus^38^ skeletal muscle^39^, and adipose tissue^40^ under normal conditions. Interestingly, we also observed that in both sexes, *GPER* mRNA and protein expressions are significantly reduced in the stomach and colon of *db/db* mice compared to the WT control (particularly in *db/db* males) (Figs. 1, 2 and 3), indicating that T2D could be a risk factor for the downregulation. Despite the dearth of experimental data on GPER expression in diabetic models, a few studies have reported the downregulation and upregulation of *GPER* mRNA and protein expression in other murine tissues/cell line models of T2D^42^. For instance, *GPER* mRNA expression was downregulated in human liver cell line models of T2D^40^, and its protein expression was downregulated in the heart of T2D rats^43^. In other studies, *GPER* mRNA and protein expressions were found to be upregulated in the vascular smooth muscles of female *db/db* mice and its protein content was upregulated in the pancreatic islets of male rats^44,45^. These inconsistent GPER expression patterns in various tissues of diabetic models imply that there’s not necessarily either upregulation or downregulation of GPER under T2D, and so more studies are needed to determine the mechanisms that determine the direction of the regulation. This should enable researchers to draw logical conclusions on how T2D impairs GPER expression and signaling in various tissues.

The reduction of GPER expression in the gastric smooth muscle of male and female db/db mice led to a slowing of the gastric emptying thereof. If GPER, as we suspect, plays a pivotal role in GI motility, it would be logical that animals with a condition that leads to lower GPER expression (T2D) would have affected GI motility which, in this case is manifested in the form of slower gastric emptying, which predisposes the animals to gastroparesis. Gastroparesis, in turn, is a condition well known to affect the female and diabetic population prominently^11,14,41,46–48^.

Similarly, the difference in physiology also affects the different outcomes for the same problem (GPER reduction related to diabetes) in the colons of male and female mice. In this case, sex-related differences in GI motility mechanisms are not unusual. For instance, GI motility is mediated partially by testosterone and depends on androgen receptors found in the enteric neurons that innervate the colon of male mice specifically^49^. When stimulated by testosterone, the enteric neurons lead to the contraction of the smooth muscle of the colon of male mice. Meanwhile, GPER facilitates the relaxation of the GI smooth muscle in male mice. However, the androgen receptors are absent in the smooth muscle of the colon of female mice^49^, and thus, testosterone would not favor contraction in females, even if testosterone was supplemented. This makes smooth muscle GPER expression and activation far more crucial in females than in males for GI motility, and would create differences between the outcomes of male and female mice if it were up or downregulated.

Higher CpG islands imply a higher number of opportunities for methylation and gene silencing, which may have an impact on the complications and symptomatology of T2D. Ahmed et al.^27^, found that CpG counts for pancreatic islets of T2D humans were increased compared to non-diabetic patients, not being apparently correlated with lower GLP-1R expression. Our study, however, found higher CpG counts at the promoter sequence of *GPER* in the *db/db* mice compared to the WT, and it related to lower *GPER* mRNA expression and GPER protein expression, indicating that the elevated CpG counts may not be an inconsequential artifact of T2D. Even among the *db/db* mice, the higher CpG counts found in the gastric smooth muscle of males seemed to result in lower *GPER* mRNA levels, and lower GPER protein levels when compared to their female counterparts.

DNA methylation, the deposition of methyl groups on CpG islands mostly located in the promoter sequence of genes, is the most widely studied mechanism of gene suppression^45,50–57^. Mechanically, gene suppression associated with DNA methylation usually involves promoter hypermethylation that is caused by the upregulation of DNMTs^24,57^. Here, we identified differentially methylated regions (hypermethylated regions 1 and 2 and hypomethylated region 3) among the nine regions of interest (regions 1–9) we targeted in the *GPER* gene promoter sequence found upstream of the gene’s transcription start site (TSS) located in chromosome 5. Regarding total CpG methylation, hypermethylation was only found to be significantly higher in the stomach of *db/db* males and the colon of *db/db* females compared to their WT controls. This is consistent in the female *db/db* mice, with elevated protein levels of DNMT3B in the colon when compared to WT (male and female) and *db/db* males; however, such a significant difference was not found in *db/db* males when compared to WT. Regardless of hypermethylation levels, there were reduced mRNA and protein expression levels observed in the *db/db* groups compared to their WT controls in the same tissues. DNMT3A and B are called de novo DNMTs and are known to establish DNA methylation in an unmethylated DNA from the early developmental stages of mammals^54,58^. These observations, therefore, imply that GPER downregulation observed in the stomach and colon of *db/db* mice compared to their WT counterparts could result from DNMT3-mediated promoter hypermethylation triggered by diabetic conditions in the *db/db* mice; however, this doesn’t seem to be the only mechanism behind the outcome.

Advances in epigenetic studies suggest that DNA methylation usually engages in crosstalk with histone modifications to regulate chromatin accessibility^59^. This observation is supported by the fact that several DNA methylation readers are also writers and erasers of histone marks while a lot of histone modification readers are known to recruit DNMTs to their target genes^24,58,60^. Repressive histone marks such as H3K9me3 and H3K27me3 methylation are associated with hypermethylated promoters and their enrichment decreases with a decrease in promoter methylation^61^. In contrast, active histone marks such as H3K4me3 and H3K27ac are hallmarks of transcriptionally active genes that associate favorably with hypomethylated promoters and their enrichment decreases the extent of promoter methylation of nearby genes^29^. Moreover, the ADD domain found in DNMT3A/B is known to interact with unmethylated H3K4 and then catalyze the methylation of CpG islands of nearby promoters leading to their silencing^24^. Hence, an increase in H3K4 methylation impedes DNMT3A/B binding and prevents the nearby promoters from methylation^58^.

Our data reported lower relative abundances of H3K4me3 and H3K27ac in the stomach and colon of the *db/db* female mice compared to the WT controls (Figs. 9 and 10). In male *db/db* mice, H3K4me3 and H3K27ac were found to be lower when compared to WT in the colon; H3K4me3 was significantly lower in the stomach, while the H3K27ac was not significantly lower (Figs. 9 and 10). This is, to our knowledge, the first report of H3K4me3 and H3K27ac levels in the gastric and colonic smooth muscle of mice.

Similarly, the enrichment of these marks around the *GPER* promoter as indicated by ChIP sequencing is also significantly reduced in the stomach and colon of all the *db/db* female and male mice compared to their respective WT controls except for the H3K4me3 which shows a slight but insignificant increase in the male colon of *db/db* mice compared to WT (Fig. 12b). This may explain why total promoter methylation and DNMT3A/B levels are not significantly different between the *db/db* and WT mice in the male colon since the little enrichment of the H3K4me3 could abolish DNMT3A/B binding and prevent promoter hypermethylation. It is noteworthy that H3K27ac is a prerequisite for writing H3K4me3 as inhibiting the former abolishes the writing of the latter^62^; hence, both marks are required for gene activation. We may, therefore, infer from these observations that the reduced enrichment of the global H3Ac and specifically the H3K27ac mark around the *GPER* promoter in the diabetic mice impairs the writing of the H3K4me3 mark, and thus, encourages the binding of DNMTs to the unmethylated H3K4 tails around the *GPER* promoter leading to its suppression by hypermethylation.

In-vivo data is invaluable to determine whether presupposed effects of gene suppression play out as expected in real life as suggested by the in-vitro experiments. The smooth muscle-specific *GPER* KO mice (Fig. 13a) were produced to help elucidate causal links between suppressed GPER expression and the gastrointestinal symptomatology of conditions that lead to reduced or suppressed GPER expression. This model should be helpful for numerous conditions including diabetes, considering our findings about the GPER suppression in the gastric and colonic smooth muscles of *db/db* mice. The *GPER* mRNA levels found in the smooth muscle-specific *GPER* KO mice (Fig. 13b–e) are comparable to those found in the *db/db* mice in this study, which suggests that further research is needed to determine which specific cells are responsible for the remaining *GPER* mRNA in *db/db* mice in the gastric and colonic tissue.

Our findings about the smooth muscle-specific *GPER* KO mice indicate the importance of sexual differences in defecation patterns and their relationship with GPER expression. The fecal pellet output (total and pattern) of the smooth muscle-specific *GPER* KO mice suggest that:

1. Sex, age, and GPER levels influence the pattern of the number of pellets produced per day (Fig. 14). This may be related to the hormonal changes of the estrous cycle in both WT and smooth muscle-specific *GPER* KO mice.
2. The alterations of the patterns include, on average, lower numbers of pellet production for the smooth muscle-specific *GPER* KO mice compared to the WT (Fig. 14a and b).
3. Smooth muscle-specific *GPER* KO mice have altered gastrointestinal motility patterns, in terms of the numbers of fecal pellets per day, which are distinct from their WT counterparts (Fig. 14c and d).
4. The absence of GPER in female smooth muscle-specific *GPER* KO mice may help explain a defecation pattern that is more consistent with the smooth muscle-specific *GPER* KO male than the WT female, even if the discrepancy with the WT female is not complete (Fig. 14b and d).

It may be worth noting that the reason for using the Tagln-cre genotype was to avoid a genotype that could express Cre in the germline, and thus to help preserve the model through reproduction for the future.

**Figure 15:**
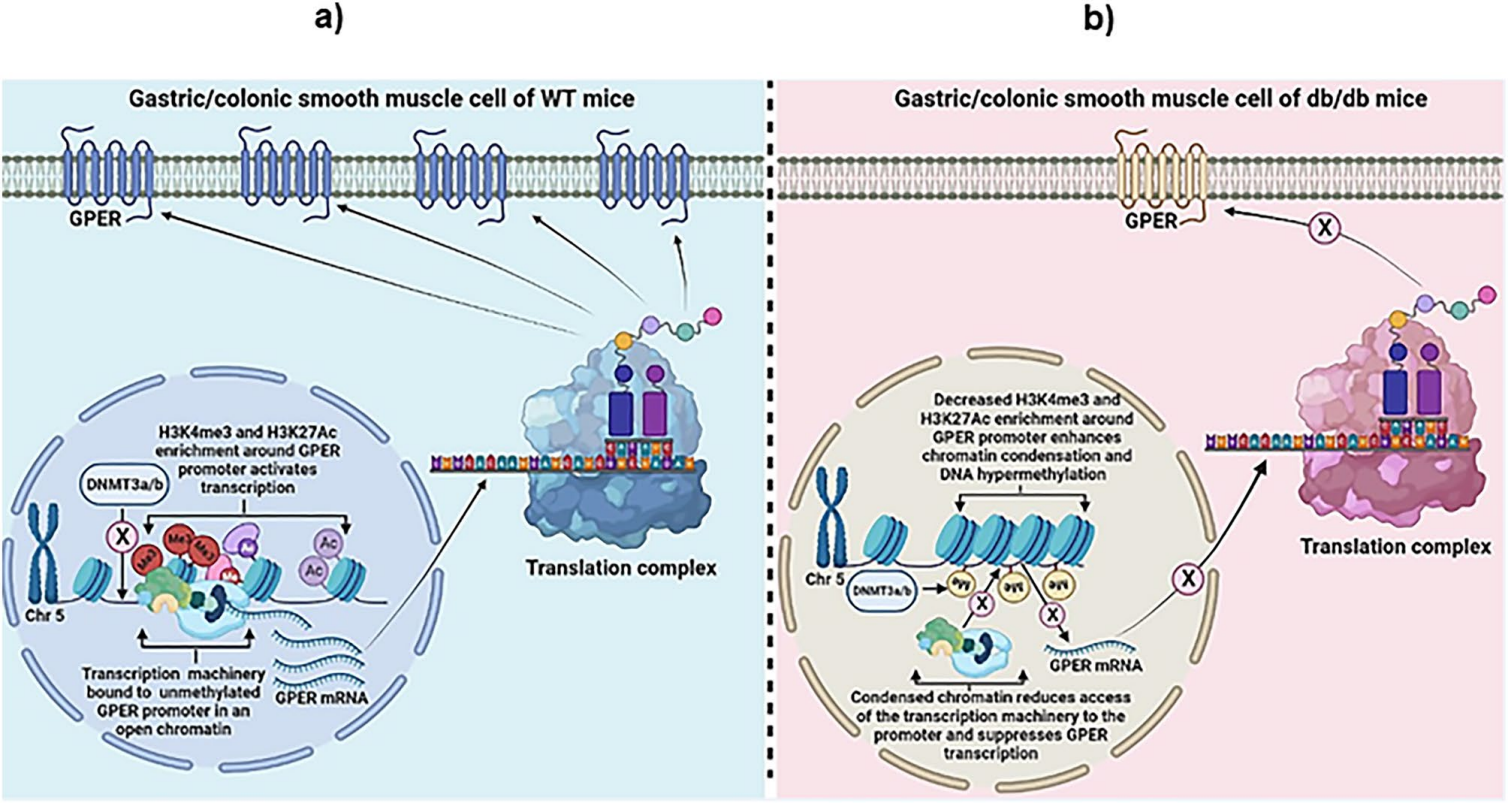
Epigenetic mechanism of GPER downregulation the gastrointestinal smooth muscle of diabetic mice. GPER protein expression is regulated by a series of molecular mechanisms that involve histones and histone tails (histone modification, namely methylation and acetylation ratios), methylation or acetylation of the promoter region of *GPER,* the translation complex, and the cell membrane. (**a**) Normal *GPER* expression in WT mice gastrointestinal smooth muscle. There is a certain degree of methylation and acetylation of the histone tail that regulates the expression of *GPER*. This allows for the unfolding of the DNA, which allows for transcription and later translation of the *GPER* mRNA. This is permitted by scarce methylation of the promoter region of *GPER*. After translation, the receptor is expressed on the cell membrane. (**b**) Abnormal *GPER* expression in *db/db* mice gastrointestinal smooth muscle. The H3K43me (methylation) and H3K27AC (acetylation) ratio of the histone tails of diabetic mice is decreased compared to that of the WT mice. Moreover, the total CpG counts (opportunities for methylation) in the promoter region of *GPER* are more abundant, which was accompanied by more instances of methylation, even though not a higher methylation ratio compared to WT. This hypermethylation limits the potential binding of the RNA polymerase, and thus limits the transcription step. With limited *GPER* mRNA expression, translation of *GPER* also occurs less frequently, leading to a lower GPER protein expression on the cell membrane.

In a nutshell, we have shown here that females generally exhibit higher GPER expression than males in the stomach and colon of WT and *db/db* mice, and T2D significantly impairs GPER expression in both males and females. We have also shown that crosstalk involving DNA methylation and histone modifications might be involved in the downregulation of GPER in the *db/db* mice (Fig. 15). Moreover, previous studies suggest that GPER expression and signaling are associated with smooth muscle relaxation, GPER activation is related to GIM and behavior associated with visceral pain in the GIT and correlates with upper GIT disorders such as GERD^22,63,64^. T2D is also a major risk factor for the upper and lower GIT disorders such as GERD, gastroparesis and enteropathy all of which are associated with diabetic neuropathy^9,64^. Furthermore, the higher prevalence of GIT disorders such as gastroparesis and GERD observed mostly in pregnant, premenopausal, and post-menopausal women with hormone replacement therapy has been linked with estrogen-mediated GPER signaling^22^. Constipation is also a common complication of diabetes, pregnancy and postpartum, and may be associated with the hormonal changes that are associated with pregnancy and childbirth (characterized by high progesterone and moderate to low estrogen levels), as well as GPER expression deficiencies during diabetes^65,66^. This, therefore, may also have to do with the aberrant GPER expression patterns we observed in *db/db* female mice. However, the biggest puzzle that remains unsolved in this research is that while previous data suggest that GPER signaling could worsen GIT disorders in non-diabetic conditions, we observed a significant downregulation of GPER expression under diabetic conditions which may either be of therapeutic or pathogenic relevance. Hence, this mystery could only be unveiled by studying the role of GPER signaling in diabetes-induced GIT disorders in further studies involving miRNAs.

What helps give a clearer case for GPER as an important factor for GI motility is that non-diabetic smooth muscle-specific *GPER* KO mice, male and female, showed slower GI transit when compared to their WT counterparts. This indicates, first, that smooth-muscle GPER is important in GI transit and secondly, that it is reasonable to expect slower GI transit in animals with conditions that lead to lower smooth-muscle GPER expression, as we found in the db/db mice.

Smooth muscle GPER, as shown by the smooth muscle-specific *GPER* KO mice, has an impact on male GI transit, with lower levels leading to slower transit. Males, however, have no ovaries to produce estrogen to activate the GPER receptors in the GI tract. The solution in male physiology is to convert testosterone into estradiol^67^, which in turn can activate the GPER receptor. Because of this, GPER and estrogens still help mediate gastroparesis and constipation in males. The mechanisms of the difference in GPER expression between WT and db/db male mice are not clear, but they may be influenced by epigenetic mechanisms, testosterone levels, their capacity to convert testosterone into estradiol, or the rate at which the estradiol is excreted in the urine.

There are some limitations to this study: we did not check the stage of the estrous cycle of the female mice involved. These hormonal changes may impact the results in terms of GPER expression and GIT. The number of female WT mice for the pellet count was small (2). Even though fecal pellet counts were compared between WT and smooth muscle-specific *GPER* KO mice, fecal pellet counts were not performed in db/db mice of either sex.

## Conclusion

*GPER* mRNA and protein expression in the gastric and colonic smooth muscles is influenced by DNA hypermethylation and histone modification and is thus reduced in both male and female *db/db* mice, where the hypermethylation is most pronounced. However, the H3K4me3 and H3K27ac modifications were not equal across the sexes and the tissues, which indicates non-uniform changes in epigenetic modification of the different parts of the digestive tract exposed to one common systemic condition. This underlines the importance of GI health studies being differentiated by the sex of the patient to be able to assess better the conditions in human patients in different stages of their reproductive age and function.

## Materials and methods

### Animals

Approximately 10–12 weeks-old age-matched male and female, Wild type (WT) (C57BLKS/J) and type II diabetic *db/db* mice (BKS.Cg.DOCK7m^+/+^ Lepr^db^/J) were purchased from Jackson Laboratories and housed on a 12 h/12 h dark/light cycle and allowed food and water ad libitum in the animal facility provided by the Division of Animal Resources, Tuskegee University. All procedures were approved by the Institutional Animal Care and Use Committee at Tuskegee University before the start of any experiments. All methods were performed in accordance with the relevant guidelines and regulations. The study is reported in accordance with ARRIVE guidelines.

### Preparation of gastric and colonic smooth muscle strips

Mice were anesthetized by CO_2_ inhalation/asphyxiation followed by cervical dislocation. The stomach and colon were removed, and the mucosa was scraped from gastric and colonic muscle layers. Smooth muscle strips from gastric and colonic smooth muscle layers were used for genomic DNA extraction for bisulfite assay and ChIP assay, nuclear and total extractions for DNMT3A/B assay and histone K4 try methylation (H3K4me3), and histone K27 acetylation (H3K27AC), RNA isolation, qPCR, and western blot studies.

### Generation of smooth muscle-specific *GPER* Knockout (*GPER* KO) mice

Homozygous GPER floxed mice were procured from Wake Forest University and subsequently bred at the College of Veterinary Medicine Small Animal Facility at Tuskegee University to maintain the GPER flox genotype. The F1 generation was then bred with heterozygous Tagln-Cre (also known as SM22α-Cre) mice obtained from Jackson Laboratories, which express the Cre recombinase under the control of the mouse smooth muscle transgelin promoter. Genotyping of F2 mice was performed by the CelPlors laboratory using the following primers:

- Cre F: 5′ GCGGTCTGGCAGTAAAAACTATC 3′;
- Cre-R: 5′ GTGAAACAGCATTGCTGTCACTT 3′;
- GPERF:5′GAACCCACAGCTCTCTTGTGTGC3′;
- GPER-R:5′GAGTGTGTGGTGTGGGAATTTGAGG 3′.

This analysis revealed the presence of heterozygous GPER flox/GPER mice carrying the Cre gene. Subsequently, these GPER flox/GPER Cre mice were further bred with homozygous GPER flox mice, resulting in the production of some F3 mice that were homozygous for GPER flox and heterozygous for the Cre gene. This breeding strategy yielded smooth muscle-specific GPER knockout mice with a GPER flox/GPER flox Cre genotype.

### RNA isolation from male and female WT and *db/db* mice; WT and smooth muscle specific *GPER* Knockout (*GPER* KO) mice gastric and colonic smooth muscle

Gastric and colonic smooth muscle from wild type (WT) and type II diabetic mice (*db/db*); WT and smooth muscle specific *GPER* KO were homogenized in 0.5–1 ml of TRIzol and collected homogenized solution in a microfuge tube, followed by 0.2 ml of RNAse/DNAse treated water and chloroform. The mixture was incubated at room temperature for 15 min before being centrifuged at 12,000 g for 15 min at 4 °C. Transferring the top aqueous phase to a fresh tube, 0.5 ml of isopropanol was added. The mixture was incubated at − 80 °C overnight before being centrifuged at 12,000 g for 15 min at 4 °C. The supernatant was discarded, and the pellet was resuspended in 1 ml of cold ethanol (75%). The mixture was centrifuged again at 12,000 g for 15 min. The supernatant was aspirated and air-dried for 15 min at room temperature. The concentration of total RNA was evaluated using spectrophotometry after it was dissolved in 0.1% diethylpyrocarbonate (DEPC)-treated water^68,69^.

### Quantitative real time-PCR (qRT-PCR) analysis

RNA from each preparation was reverse transcribed in a 20 ml reaction volume using the High-Capacity cDNA Reverse Transcription kit. TaqMan PCR Mastermix performed qRT-PCR on cDNA samples using TaqMan-specific primers for *GPER* (Mm01194815_m1) and beta-actin (Mm02619580_g1). The target gene’s starting copy number calculates the threshold cycle parameter, defined as the fractional cycle at which the fluorescence produced by probe cleavage exceeds a fixed threshold above the baseline. The data were quantified and normalized to beta-actin using 2^−ΔΔCt^ method. The results are a fold change expression between *db/db* and WT mice samples. All PCR reactions were performed in an ABI QuantStudio 5 or 7500 Fast Real-Time PCR system^68,69^.

### Western blot analysis

WT and *db/db* muscle strips were solubilized in a Triton X-100-based lysis buffer containing protease and phosphatase inhibitors (100 g/ml PMSF, 10 g/ml aprotinin, 10 g/ml leupeptin, 30 mM sodium fluoride, and 3 mM sodium vanadate). The protein concentrations in the supernatant were determined using a Bio-Rad Dc protein assay kit after centrifuging the lysates at 20,000 g for 10 min at 4 °C. SDS/PAGE was used to separate equal amounts of proteins, which were then transferred to a PVDF membrane. Blots were blocked in 5% (w/v) nonfat dried milk/TBS-T [Tris-buffered saline (pH 7.6) plus 0.1% for 1 h before incubating with GPER (Novus Biological; 1:1000) or b-Actin (Sigma; 1:5000) antibodies in TBS-T plus 5% (w/v) nonfat dried milk. After 1 h of incubation in TBS-T plus 1% (w/v) nonfat dried milk with the horseradish-peroxidase-conjugated corresponding secondary antibody (1:5000), immunoreactive proteins were visualized using the Pierce ECL Western Blotting substrate kit (Thermo Fisher). TBS-T was used for all washing steps. The GE Amersham 680 Imaging system’s enhanced chemiluminescence reagent detection identified the protein bands^68,69^.

### Targeted bisulfite sequencing of male and female gastric and colonic smooth muscle from WT and *db/db* mice

The bisulfite assay was used to analyze the *GPER* DNA methylation status of cytosine residues in a DNA molecule in the gastric and colonic smooth muscle of male and female WT and *db/db* mice. The MethylCheckTM Service was used for processing and analyzing the samples. Assays were designed to target CpG sites in the *GPER* promoter specified region of interest (ROI) using primers designed with Zymo Research’s proprietary sodium bisulfite converted DNA-specific primer design tool, Rosefinch. The primers were designed to minimize amplification bias by synthesizing ordered primers with a pyrimidine (C or T) at the CpG cytosine in the forward primer and a purine (A or G) at the CpG cytosine in the reverse primer. The primers were validated using Real-Time PCR with bisulfite-converted control DNA, and the PCR product was confirmed by DNA melt analysis. After primer validation, the samples were bisulfite converted using the EZ DNA Methylation-LightningTM Kit (Zymo Research, D5030) following the manufacturer's instructions. All samples were then amplified using ROI-specific primer pairs with limited amplification cycles. The resulting amplicons were pooled, barcoded, and purified using the DNA Clean & Concentrator-5TM kit. The samples were then prepared for massively parallel sequencing using an Illumina MiSeq V2 300 bp Reagent Kit and paired-end sequencing protocol as directed by the manufacturer. Sequence reads were identified using standard Illumina base-calling software and analyzed using a proprietary analysis pipeline written in Python by Zymo Research. The methylation level of each sampled cytosine was calculated by dividing the number of reads containing a C by the total number of reads containing a C or T.

### GPER promoter sequence

“CCACCTTGGTTGTACACGGTCTAGAACTAGAGACAGAGCTGACCCCTGGGTAGAGGGACATTAGCCCTAGCTAACATTAGAGTAGAAAACACACCTGGATTCCTAATTTCTGGTCAAAATGCCGGCTACTTGTAGACTGTATTTCCCACTTCGAGCATCCCTAGGAAAACTATAGCTTACATTTGCTGTGTGACTGCAGTCCTACCATGCTGATTGAGGAAACTCAGTGTTCAGTTTTAAAGCCAGCACAAGCTAAAACAGGCAAGGGTATCATTGCTTCAACAAATGAGGAAGGATTCTTACCTAAAAGGTAAACAATATTATAATCCTTTCACACTTTAAATAAATGTGTCAGTGGGTGAAAATCAGCAGTCACACTGGAAACTTCCATAAAATACACATCCAGCAGGGTCGTTTTCACTGTCTACATGTGGGAGGAAAAAAACTGCCAGCAAAAAAATGGTTAATGCTGGCCTTAAAGGGAGGCTGGCCAGAGCCCAGTGAGTAGGCTTGGGAAGTCTATAAAGGAGGCGCTGTGCCAAGGGGGCCAGACGCTGCTGGACGGCCACAGGCATCCATCCCCAGGCATCGGGCGGGTGCTTCTGTTCCTCTCCTGCTGGGTCCCTGCTGGGCACCGTCCCCAAAGTGCTGCAAGTCCAGGGTCCATCCCTGGAGCAAGCTCCAGGAGCACCTCCAGCAG”.

### DNA methyltransferase 3A and 3B analysis of male and female WT and *db/db* mouse gastric and colonic smooth muscle

DNA Methyltransferase 3A & 3B Assay Kit (Epigentek, Brooklyn, NY) was employed, using nuclear extracts from gastric and colonic smooth muscle from WT and *db/db* male and female mice. Ten micrograms of nuclear extracts were incubated with capture reagent and assay buffer for 2 h at 37 °C and then exposed to the affinity antibody for 60 min and the detection antibody for 30 min at room temperature. Absorbance was determined using a microplate spectrophotometer at 450 nm, and DNMT percent change was calculated according to the following formula: (Treated (*db/db*) sample OD−blank OD)/(Untreated (WT) sample OD-Blank OD))*100, according to the manufacturer's instructions. Protein standards of known concentration (30 ng, 20 ng, 10 ng, and 2 ng) were included to generate a standard curve. Results were expressed as DNMT3A or DNMT3B percent change.

### Nuclear extraction of male and female gastric and colonic smooth muscle from WT and *db/db* mice

The EpiQuick Nuclear Extraction Kit (Epigentek) was used to extract nuclear proteins according to the manufacturer’s protocol. Approximately 20 mg of smooth muscle tissues from the stomach and colon were minced and homogenized in a dounce homogenizer with NE1 buffer. The samples were collected and centrifuged for 10 min at 12,000 rpm. The nuclear extract was made by adding two volumes of NE2 buffer to the pellet and sonicating it before centrifuging it at 14,000 rpm for ten minutes. Nuclear extracts were kept at − 80 °C until they were used. The Quick Start Bradford Protein Assay was used to determine the protein concentration of the nuclear extract (BioRad, Hercules, CA).

### Total histone extraction of male and female gastric and colonic smooth muscle from WT and *db/db* mice

Total histones were extracted using the EpiQuick Total Histone Extraction Kit according to the manufacturer’s protocol. Approximately 20 mg of smooth muscle tissues from the stomach and colon were minced and homogenized in a Dounce homogenizer with a pre-lysis buffer. The pre-lysate mixture was centrifuged for one minute at 10,000 rpm. The total histone extractions were made by adding three volumes of lysis buffer to the pellet. The lysates were centrifuged at 12,000 rpm for 5 min, and 0.3 volumes of balanced DTT buffer were added to the supernatant. Total histones were kept at − 80 °C until they were used. The Quick Start Bradford Protein Assay was used to determine the protein concentration of the nuclear extract (BioRad, Hercules, CA).

### Histone 3 lysine 4 trimethylation (H3K4Me3) and histone 3 lysine 27 acetylation (H3K27AC) assay of male and female gastric and colonic smooth muscle from WT and *db/db* mice

Histone 3 Lysine 4 Trimethylation and Histone 3 Lysine 27 Acetylation were measured by EpiQuick H3K4Me3 and H3K27AC assay kits, using total histone extracts from gastric and colonic smooth muscle from WT and *db/db* male and female mice. Briefly, one microgram of histone proteins was used to measure the levels of histone proteins. In these ELISA-based assays, methylated and acetylated histones were captured by their respective, specific antibodies. They were measured using a detection antibody and color development reagent and quantified by colorimetric analysis at 450 nm using a Promega GloMax absorbance plate reader. H3K4me3 and H3K27AC percentage was calculated according to the following formula: (Treated (*db/db*) sample OD−blank OD)/(Untreated (WT) sample OD-Blank OD))*100, according to the manufacturer’s instructions. Protein standards of known concentration (1.5, 3, 6, 12, 25, 50, and 100 ng/µl) were included to generate a standard curve. Results were expressed as H3K4Me3 and H3K27ac percentages.

### Protein cross-linking and chromatin immunoprecipitation of male and female gastric and colonic smooth muscle from WT and *db/db* mice

We performed a ChIP assay using a modified version of the protocol described by Fomsgaard et al.^70^. Here we write the protocol, including the modifications: Before the chromatin immunoprecipitation assay, proteins were cross-linked to the DNA following a specific protocol. In brief, once dissected, gastric and colonic smooth muscle strips were placed immediately in liquid nitrogen and followed by − 80 °C until the next step. Gastric and colonic smooth muscle strips were cut into small pieces and placed in a 15-ml centrifuge tube containing DPBS (pH 7.4) with 1% formaldehyde and kept at room temperature (RT) for 8–10 min. The reaction was stopped by the addition of 10 × glycine to a final concentration of 0.125 M and 5-min incubation at RT. The tissue was washed four times in cold phosphate-buffered saline (PBS) containing protease inhibitors (Complete; Roche, Branchburg, NJ) and homogenized in ice-cold cell lysis buffer (10 mM NaCl, 0.2% Nonidet P-40, and 10 mM Tris–HCl; pH 8.0) with protease inhibitor cocktail II. The nuclear fraction was lysed in ice-cold nuclear lysis buffer (10 mM EDTA, 1% SDS, and 50 mM Tris–HCl; pH 8.0) with protease inhibitor cocktail II and sonicated on ice with the use of an Active motif sonicator. The lysate was centrifuged to remove insoluble material and then diluted 1:10 in ChIP dilution buffer (167 mM NaCl, 0.01% SDS, 1.1% Triton X-100, 1.2 mM EDTA, and 16.7 mM Tris–HCl; pH 8.1) to a final volume of 1.0 ml. Primary antibodies were added to diluted lysates and incubated at 4 °C for 12 h with 15 ml of fully suspended protein A/G magnetic beads (EMD Millipore). The following primary antibodies were used: acetyl-histone H3 (EMD Millipore; 06-599, 1:200), acetyl-histone H3 (Lys27) (Active Motif; 39133, 10 mg), tri-methyl-histone H3 (Lys4) (Active Motif; 39159, 1: 200). The chromatin/immune complexes were washed with low salt immune complex wash buffer (150 mM NaCl, 0.1% SDS, 1% Triton X-100, 2 mM EDTA, and 20 mM Tris–HCl; pH 8.1), high-salt immune complex wash buffer (500 mM NaCl, 0.1% SDS, 1% Triton X-100, 2 mM EDTA, and 20 mM Tris–HCl; pH 8.1), LiCl immune complex wash buffer (0.25 M LiCl, 1% NP-40, 1% deoxycholic acid sodium salt, 1 mM EDTA, and 10 mM Tris–HCl; pH 8.1), and TE buffer (1 mM EDTA and 10 mM Tris–HCl; pH 8.0). The chromatin/immune complexes and input DNAs were reverse cross-linked by incubation with ChIP elution buffer (1% SDS and 0.1 M NaHCO3) containing 0.1 mg/ml proteinase K for 2 h at 62 °C. DNA was purified by using spin columns (EMD Millipore). Input and immunoprecipitated DNAs were subjected to qPCR using the promoter using promoter region of *GPER* primers F:5′CCCAGTGAGTAGGCTTGGGAA 3′ and R: 5′AGCACTTTGGGGACGGTG 3′. All assays included non-immune IgG to control for the specificity of each antibody used. All reactions were confirmed to generate a single PCR product by gel melting curve analysis. Data are shown as fold enrichment to IgG for male and female wild-type versus *db/db* mice, depending on the antibodies tested.

### Assessment of fecal output

We examined fecal pellet production in both male and female mice, comparing wild-type (WT) and smooth muscle-specific *GPER* Knockout (*GPER* KO) mice. Each mouse was housed individually in a metabolic cage for a continuous period of 30 days, with a specified quantity of food and water provided ad libitum. Every 24 h, fecal pellets were collected from each mouse, and the number of pellets was manually recorded over the course of the 30-day period.

### Statistics and reproducibility

All the results were analyzed by A two-way analysis of variance (ANOVA) with multiple comparison tests. mRNA expression results were analyzed by Student’s t-test for paired and unpaired values. All statistical analysis and graphs were done in GraphPad Prism 10 (GraphPad Software, Inc., USA). Data are presented as the mean ± standard error of the mean (SEM) with p < 0.05 considered statistically significant. For each statistical analysis, data derived from at least three independent experiments were used. Experiments were considered independent when they were derived from distinct samples and thus reflected biological replicates. Sample sizes are indicated for all experiments in the respective figure legends. The results should be reproducible so long as the same methods, including the same mouse strains, are used, and the mice are kept in a controlled environment.

## Data Availability

Data are available in the Gene Expression Omnibus (GEO) datasets. Targeted bisulfite sequencing dataset is available under accession number GSE240515.
